# NSG1 promotes glycolytic metabolism to enhance Esophageal squamous cell carcinoma EMT process by upregulating TGF-β

**DOI:** 10.1038/s41420-023-01694-6

**Published:** 2023-10-23

**Authors:** Mingshu Tu, Xiaoqing Yin, Wanzhen Zhuang, Xiaoqing Lin, Yu Xia, Zhixin Huang, Yue Zheng, Yi Huang

**Affiliations:** 1 Shengli Clinical Medical College, Fujian Medical University, Fuzhou, China; 2 Department of Clinical Laboratory, Fujian Provincial Hospital, Fuzhou, China; 3 Integrated Chinese and Western Medicine College, Fujian University of Traditional Chinese Medicine, Fuzhou, China; 4 Central Laboratory, Center for Experimental Research in Clinical Medicine, Fujian Provincial Hospital, Fuzhou, China; 5 Fujian Provincial Key Laboratory of Critical Care Medicine, Fujian Provincial Key Laboratory of Cardiovascular Disease, Fuzhou, China

**Keywords:** Oesophageal cancer, Cancer metabolism

## Abstract

As a highly enriched endosomal protein within neuronal cells, NSG1 has been discovered to facilitate the process of epithelial-mesenchymal transition (EMT) in esophageal squamous cell carcinoma (ESCC). However, the precise mechanisms behind this phenomenon have yet to be elucidated. The pivotal role of transforming growth factor-β (TGF-β) in triggering the EMT and its significant contribution towards tumor metabolic reprogramming—responsible for EMT activation—has been robustly established. Nevertheless, the extent of TGF-β involvement in the NSG1-mediated EMT within ESCC and the processes through which metabolic reprogramming participates remain ambiguous. We accessed an array of extensive public genome databases to analyze NSG1 expression in ESCC. Regulation of TGF-β by NSG1 was analyzed by transcriptome sequencing, quantitative Real-Time PCR (qRT-PCR), co-immunoprecipitation (CO-IP), and immunofluorescence (IF). Additionally, cellular functional assays and western blot analyses were conducted to elucidate the effect of NSG1 on TGF-β/Smad signaling pathway, as well as its role in ESCC cell metastasis and proliferation. We validated the influence of the NSG1/TGF-β axis on metabolic reprogramming in ESCC by measuring extracellular acidification, glucose uptake, and lactate production. Our findings identify an oncogenic role for NSG1 in ESCC and show a correlation between high NSG1 expression and poor prognosis in ESCC patients. Additional research indicated TGF-β’s involvement in the NSG1-induced EMT process. From a mechanistic perspective, NSG1 upregulates TGF-β, activating the TGF-β/Smad signaling pathway and subsequently fostering the EMT process by inducing cell metabolic reprogramming—evident from elevated glycolysis levels. In conclusion, our study highlights the NSG1/TGF-β axis as a promising therapeutic target for ESCC.

## Introduction

Esophageal squamous cell carcinoma (ESCC), the most common of esophageal cancer types worldwide, ranks sixth in mortality among all malignant tumors. With its cell’s distinct invasiveness and metastasis, it often results in poor patient prognoses [1]. The global distribution of ESCC varies significantly geographically. Even with a marked increase in the incidence of esophageal adenocarcinoma (EAC) in Western countries, ESCC still accounts for over 90 percent of esophageal cancers on a global scale [2]. Particularly in China, the incidence of ESCC is continuously rising and has become one of the prominent factors contributing to deaths attributable to tumors [3]. Given the absence of early clinical signs, diagnoses for majority of ESCC patients are made at intermediate or advanced stages, culminating in a disconcerting survival rate of 15–20% after five years [4]. Thus, it becomes paramount to scrutinize the underlying mechanisms steering ESCC development and uncover novel molecular markers correlated with this disease.

Neuron enriched endosomal protein (NEEP21), also described as D4S234E or NSG1, is a protein primarily located in neuronal cells [5]. Existing research indicates that NSG1 plays a critical role on neuronal participation in synaptic trafficking and neurodevelopmental regulation [6, 7]. Nonetheless, the potential association between NSG1 and malignancies, especially ESCC, has not been comprehensively considered. Importantly, our initial study suggests NSG1 aids the epithelial-mesenchymal transition (EMT) process in ESCC cells. Further investigation, however, is needed to understand the underlying molecular mechanisms [8].

EMT is universally accepted as a dynamic developmental mechanism where epithelial cells detach from their neighboring cells and progressively transfigure into mobile mesenchymal cells [9, 10]. Therefore, the onset of EMT is principally distinguished by the reduced expression of E-cadherin, an epithelial marker, in conjunction with an increased functionality of specific core transcription factors, notably Zeb1, Snail, and Slug [11, 12]. In recent years, increasing evidences have defined the contributions of EMT-driven acquisition of mesenchymal features to tumor initiation, progression, metastasis, recurrence, and therapy resistance [13–15]. Transforming growth factor-β (TGF-β), a primary instigator of EMT [16], has a marked effect on EMT activation in many malignancies [17, 18], through the TGF-β/Smad signaling. Given this, investigating the regulatory mechanism of TGF-β and the downstream signaling may provide a promising strategy for ESCC treatment.

Metabolic reprogramming is a fundamental characteristic of most tumor cells [19]. As reported, the glycolytic process is the initial stage in the energy generation route of a normal cell, followed by mitochondrial oxidative phosphorylation [20]. Contrary to normal cells, tumor cells possess the aptitude to regulate their growth, development, and microenvironment through the modification of their metabolic profile [21–23]. Otto Warburg identified that tumor cells exhibit an elevated need for glycolysis, even when sufficient oxygen is present. These cells consume copious amounts of glucose and produce a specific quantity of lactic acid [24]. The critical role of TGF-β in reshaping metabolic processes within tumor metabolism has been comprehensively documented in recent research [25]. As an example, Nakasuka et al. reported that TGF-β, according to their study, triggers the reprogramming of amino acid metabolism, thereby enhancing the EMT process in non-small cell lung cancer (NSCLC) [26]. TGF-β has been demonstrated to instigate a shift in metabolic reprogramming from oxidative phosphorylation to glycolysis within glioblastoma, creating an immunosuppressive environment conducive to tumor growth [27]. Due to the fact that cellular metabolic re-editing is responsible for the EMT activation in many malignancies [28–30], it is reasonable that TGF-β signaling exhibits the effect on EMT process of ESCC cells by inducing metabolic reprogramming. However, up to now, whether NSG1 affects cell metabolic reprogramming and then promotes the EMT process by the crosstalk with TGF-β in ESCC remains unknown.

This study reveals that the overexpression of NSG1 triggers the EMT in ESCC cells by enhancing the TGF-β/Smad signaling pathway. Additionally, we discovered that metabolic reprogramming, which results in increased levels of glycolysis, is critical for the EMT process driven by the NSG1/TGF-β axis. The results presented in this study highlight the substantial involvement of the NSG1/TGF-β axis in the development of ESCC, implying its potential as a promising therapeutic target for individuals with ESCC.

## Results

### NSG1 is upregulated in ESCC and significantly associated with poor prognosis for ESCC patients

**Fig. 1:**
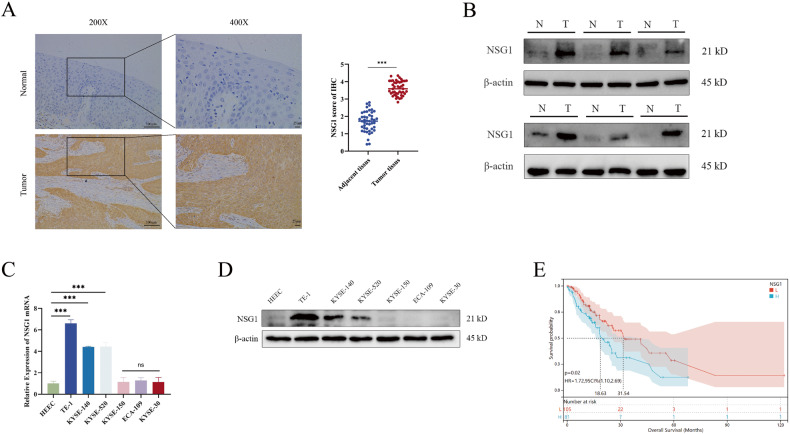
NSG1 is upregulated in ESCC and significantly associated with poor prognosis for ESCC patients. **A** ESCC and adjacent non-tumor tissues were stained for NSG1 expression by immunohistochemistry. Scale bars, 25 µm. **B** The protein level of NSG1 in the ESCC tumor (T) and adjacent normal tissues (N) was detected by western blot. **C**, **D** The expression of NSG1 in normal human esophageal epithelial cells (HEEC) and different ESCC cell lines was detected by qRT-PCR and western blot. **E** Kaplan-Meier analyses were used to estimate overall survival. Patients with high NSG1 expression had a significantly lower overall survival rate than those with low NSG1 expression (NSG1 low expression, *n* = 105 vs. NSG1 high expression, *n* = 85). Data from three independent experiments are presented the mean ± SD. *P < 0.05, **P < 0.01, and ***P < 0.001.

To assess the clinical relevance and oncogenic role of NSG1 in ESCC, we conducted a comprehensive analysis of NSG1 expression in 45 paired ESCC specimens. This included both tumor tissues and their corresponding non-tumor tissues, using the methods of immunohistochemistry and western blot analysis. Further analysis revealed an abnormal upregulation of NSG1 expression in cancerous tissues compared to the surrounding non-tumor tissues (Fig. 1A, B). Additionally, we investigated both NSG1 mRNA and protein levels across multiple ESCC cell lines. It was evident that NSG1 expression levels in TE-1, KYSE-140, and KYSE-520 surpassed those of normal human esophageal epithelial cells (HEEC) significantly (Fig. 1C, D). Furthermore, the survival curves demonstrated a significant correlation between increased NSG1 expression and poor prognosis in patients with ESCC (Fig. 1E). By analysis of the clinical characteristics of ESCC, high NSG1 level was positively associated with tumor T staging (Table 1). Consequently, these research findings suggest that the pronounced upregulation of NSG1 in ESCC tissues may contribute to the poor prognosis of ESCC patients.

**Table 1:** Relationship between NSG1 protein expression and clinicopathologic characteristics.

Parameter	Total	NSG1				*P*-value
		High expression		Low expression		
Age(y)						
<60	85	46		39		0.271
≥60	100	46		54		
Gender						
Male	159	79		80		0.976
Female	26	13		13		
Histological type						
ESCC	96	64		32		<0.001
ESCA	89	28		61		
T classification						
T1-2	74	39		35		0.406
T3-4	93	43		50		
N classification						
N0	76	42		34		0.143
N1-3	89	39		50		
M classification						
M0	153	75		78		0.143
M1	9	4		5		
Stage						
I–II	96	49		46		0.290
III–IV	65	28		37		

### NSG1 upregulated TGF-β

**Fig. 2:**
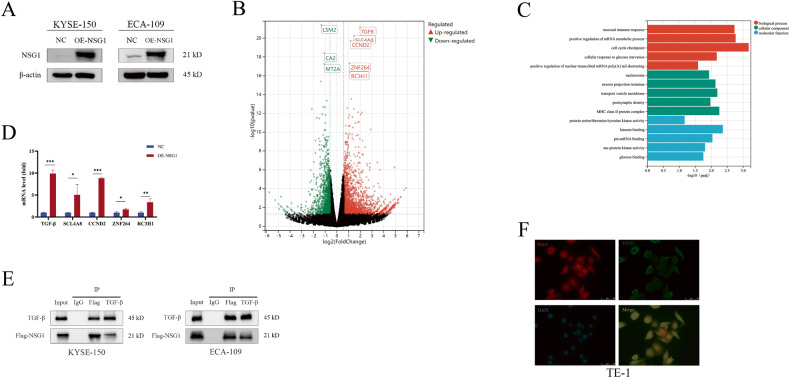
NSG1 upregulated with TGF-β. **A** KYSE-150 and ECA-109 cells were transfected with NSG1. The protein expressions were analyzed by western blot. **B** Volcano plot of differential expression genes between NC (negative controls) and NSG1 groups. **C** The top five Gene Ontology (GO) terminologies across cellular components, biological processes, and molecular functions. **D** The relative -expression of the top five upregulated genes was detected by qRT-PCR. **E** Western Blot was performed following Co-IP to validate the interaction between NSG1 and TGF-β. **F** Immunofluorescence analysis revealed the colocalization (yellow) of NSG1 (red) and TGF-β (green) within TE-1 cells. Scale bars, 50 µm.

To further understand the impact of NSG1 on ESCC development, we constructed two pairs of NSG1 overexpressing cell lines KYSE-150 and ECA-109, in which have low endogenous NSG1 expression (Fig. 2A), and further explore the NSG1 effect by transcriptome analysis. Upon NSG1 overexpression, RNA-Seq data suggests that ESCC transcriptome was dramatic changed. Among the top differentially expressed genes (DEGs), we noticed that TGF-β expression level is significantly upregulated after NSG1 overexpression (Fig. 2B). Gene ontology (GO) analysis also indicated the enrichment of glucose binding among upregulated genes, suggesting that NSG1 may have an impact on aerobic glycolysis (Fig. 2C). Although the qPCR data suggest that all five target genes were noticeably up-regulated after NSG1 transfection, the change in TGF-β was the most apparent (Fig. 2D). Given the essential role of TGF-β in EMT, we decided to concentrate our subsequent research on the cooperation between NSG1 and TGF-β. Western blot analysis disclosed a discernible augmentation in the expression levels of TGF-β within ESCC tissues as compared to adjacent precancerous counterparts (Fig. S1A). Additionally, immunohistochemical staining confirmed the upregulation of TGF-β (Fig. S1B). Clinical and pathological analyzes showed a correlation between TGF-β expression and tumor T staging (Table S1).

To investigate the potential correlation between NSG1 and TGF-β, we performed an immunoprecipitation (CO-IP) experiment in ESCC cells transfected with Flag-NSG1. It was shown that NSG1 and TGF-β exhibited reciprocal binding in KYSE-150 and ECA-109 cells (Fig. 2E). Further to this, the correlation between NSG1 and TGF-β was substantiated through immunofluorescence analysis. This showcased the co-localization of these two proteins within TE-1 cells characterized by elevated NSG1 expression (Fig. 2F). As a result, we deduced that NSG1 might interact with TGF-β and co-regulate the ESCC progression.

### NSG1 facilitates the migration and invasion of ESCC cells via the activation of TGF-β/Smad signaling pathway

**Fig. 3:**
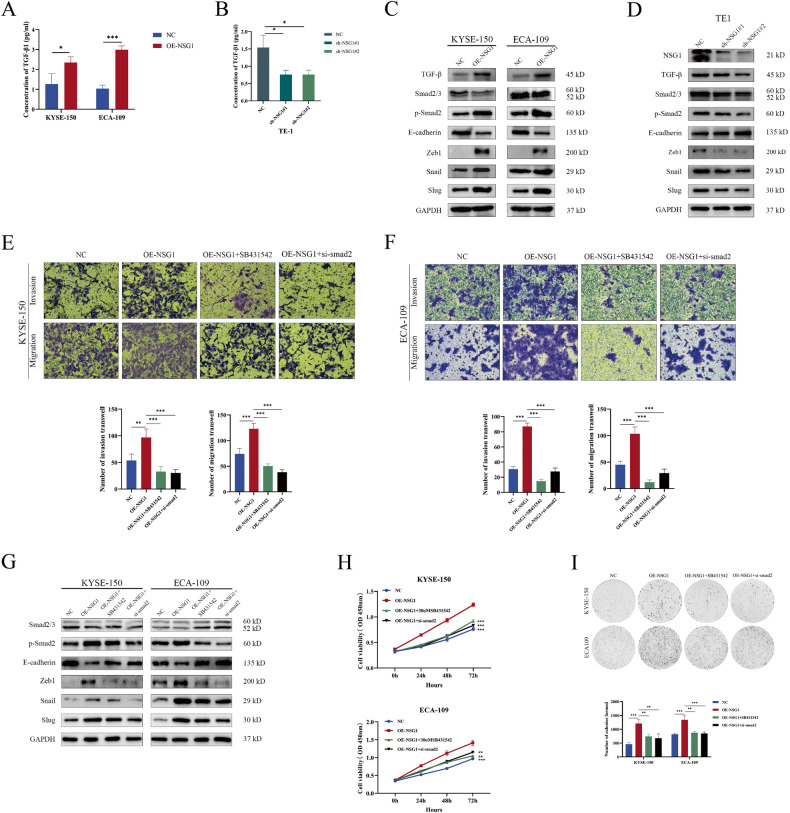
NSG1 facilitates the migration and invasion of ESCC cells via the TGF-β/Smad signaling pathway. **A**, **B** The concentration of TGF-β in ESCC cells culture supernatants were assayed by ELISA. **C**, **D** The protein levels of TGF-β, Smad2/3, p-Smad2, E-cadherin, Zeb1, Snail, and Slug in ESCC cells with overexpression or knockdown of NSG1 were determined by western blot. **E**–**I** KYSE-150 and ECA-109 cell lines were transfected with lentiviral vectors containing either the control or NSG1 genes. The cell lines that overexpressed NSG1 were then treated with either SB431542, a TGF-β inhibitor (25 µM), or si-smad2. **E**, **F** Transwell assays were performed to determine the ESCC cells’ ability for migration and invasion, 200×. **G** Protein levels of TGF-β, Smad2/3, p-Smad2, E-cadherin, Zeb1, Snail, and Slug were detected by western blot. **H**, **I** CCK-8 assays and Colony-forming assays were used to measure the proliferative ability of ESCC cells. Data from three independent experiments are presented the mean ± SD. *P < 0.05, **P < 0.01, and ***P < 0.001.

TGF-β serves as a vital cytokine instrumental to tumor progression [17]. To investigate the function of the NSG1/TGF-β axis in ESCC development, we developed two NSG1 overexpression cells: KYSE-150 and ECA-109. Additionally, shRNA was utilized to diminish NSG1 expression in TE-1 cells, which exhibit high endogenous NSG1 expression. Given the understood association between NSG1 and TGF-β in ESCC cells, we hypothesized whether NSG1 might stimulate EMT through the upregulation of TGF-β, culminating in the activation of the TGF-β/Smad signaling pathway. Subsequently, we validated the results in both cellular lysis and culture supernatant of ESCC cells overexpressing NSG1. The ELISA and western blot findings further indicated that NSG1 overexpression not only intensified the expression of TGF-β but also elevated the level of TGF-β concentration in the supernatant of NSG1 overexpression culture (Fig. 3A, C). Moreover, enhanced levels of p-Smad2, Zeb1, Snail, and Slug were observed following NSG1 overexpression, but E-cadherin expression was subdued (Fig. 3C). These contrasting alterations in TGF-β/Smad signaling and EMT markers were evident in ESCC cells subjected to NSG1 knockdown (Fig. 3B, D). In order to deepen our understanding of the potential role of NSG1-induced activation of the TGF-β/Smad signaling pathway in ESCC cell invasion and migration, we exposed NSG1-overexpressing ESCC cells to SB-431542 (25 µM) - a highly selective TGF-β inhibitor, and si-smad2. Transwell assays results suggested a noticeable increase in the migratory and invasive traits of KYSE-150 and ECA-109 cells due to NSG1 overexpression. Interestingly, the implementation of the SB-431542 inhibitor and si-smad2 effectively negated this promotion (Fig. 3E, F). Consistently, the results obtained from the western blot analysis demonstrated that pretreatment with SB-431542 or si-smad2, specifically targeting NSG1-overexpressing ESCC cells, instigated the downregulation of both the TGF-β/Smad signaling pathway and EMT-associated proteins (Fig. 3G). Additionally, the use of TGF-β inhibitors (SB-431542) and si-smad2 has been demonstrated to effectively mitigate the excessive proliferation and colony formation of NSG1-induced ESCC cells (Fig. 3H, I). These results strongly illustrate that TGF-β/Smad signaling is indispensable for NSG1 to induce ESCC cell proliferation, invasion, migration, and EMT-like processes.

### NSG1 induced metabolic reprogramming in ESCC cells

**Fig. 4:**
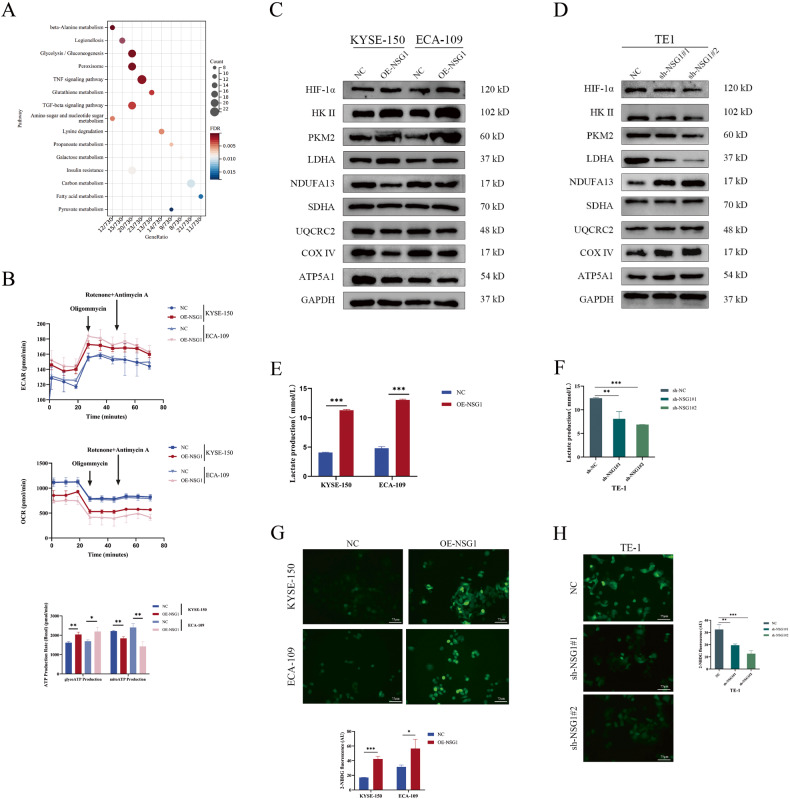
NSG1 induced metabolic reprogramming in ESCC cells. **A** The top 15 enrichment terms by KEGG pathway analysis for differential expressed mRNAs. **B** Real-time ATP rate analysis of KYSE-150 and ECA-109 cells with stable NSG1 expression. **C** Western blot assays were performed to determine the levels of HIF-1α, HK II, PKM2, LDHA, NDUFA13, SDHA, UQCRC2, COX IV, and ATP5A1 proteins in KYSE-150 and ECA-109 cells transfected with NSG1. **D** Western blot assays were performed to determine the level of HIF-1α, HK II, PKM2, LDHA, NDUFA13, SDHA, UQCRC2, COX IV, and ATP5A1 proteins in TE-1 cells transfected with NSG1-shRNA1/2. **E**, **F** Lactate production was analyzed to evaluate the LDH activity in relevant ESCC cells. **G**, **H** 2-NBDG immunofluorescence imaging were conducted to analyze glucose uptake. Scale bars, 75 µm. Data from three independent experiments are presented the mean ± SD. *P < 0.05, **P < 0.01, and ***P < 0.001.

Metabolic reprogramming is a crucial element in the development of tumors [19]. Kyoto Encyclopedia of Genes and Genomes (KEGG) data indicated that NSG1 might be involved in regulating glycolysis (Fig. 4A). Therefore, we examined the real-time ATP rate in ESCC cells to elucidate the impact of NSG1 on glycolysis. It was shown that NSG1 overexpression significantly suppresses mitochondrial oxidative respiration, while increasing glycolysis in ESCC cells (Fig. 4B). To substantiate our hypothesis, we evaluated the expression levels of proteins associated with glycolysis and the complexes in the inner mitochondrial membrane following NSG1 overexpression. Western blot analysis indicated that NSG1 overexpression notably elevated the expression levels of proteins such as the hypoxia factor-1α (HIF-1α), hexokinase II (HK II), pyruvate dehydrogenase kinase 1 (PDK1), and lactate dehydrogenase-A (LDHA). However, NSG1 overexpression lowered the expressions of the mitochondrial respiratory chain proteins NDUFB13 (complex I), UQCRC2 (complex III), COXIV (complex IV), and ATP5A1 (complex V), while the SDHA (complex II) expression was not affected (Fig. 4C). Conversely, NSG1 knockdown upregulated the expression of mitochondrial respiratory chain proteins while downregulated the expression of glycolysis-related proteins (Fig. 4D). Lactate production is considered as a critical factor in assessing the Warburg effect [31]. Thus, we observed that NSG1 stimulated lactate production in KYSE-150 and ECA-109 cells, whereas NSG1 knockdown reduced lactate accumulations (Fig. 4E, F). Additionally, results from 2-NBDG fluorescence labeling trials demonstrated that overexpression of NSG1 increased fluorescence intensity, thus promoting glucose uptake. Conversely, TE-1 cells exhibited opposing outcomes when NSG1 was suppressed (Fig. 4G, H). These findings indicated that NSG1 induces the metabolic reprogramming of ESCC cells and promotes glycolysis pathways.

### TGF-β is required for NSG1-mediated glycolysis activation in ESCC cells

**Fig. 5:**
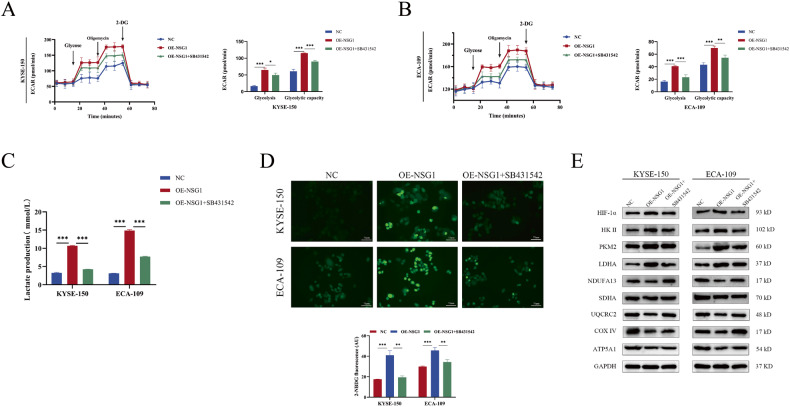
TGF-β is required for NSG1 induced glycolysis activation in ESCC cells. **A**–**E** KYSE-109 and ECA-109 cells were transfected with NSG1 and treated with SB-431542 (25 µM). **A**, **B** The extracellular acidification rate (ECAR) was determined after injecting glucose, oligomycin, and 2-DG. **C**, **D** Lactate production and 2-NBDG immunofluorescence were conducted to analyze the LDH activity and glucose uptake. Scale bars, 75 µm. **E** Western blot was performed to detect the glycolytic-related proteins and respiratory chain complexes-related proteins, such as HIF-1α, HK II, PKM2, LDHA, NDUFA13, SDHA, UQCRC2, COX IV, and ATP5A1. Data from three independent experiments are presented the mean ± SD. *P < 0.05, **P < 0.01, and ***P < 0.001.

To define the role of TGF-β on NSG1-induced glycolysis of ESCC cells, we suppressed TGF-β expression in cells with NSG1 overexpression. ECAR data showed that NSG1 overexpression boosted basal glycolysis and glycolytic capability; however, SB-431542 reduced TGF-β expression and reversed this effect (Fig. 5A, B). Moreover, TGF-β inhibition by SB-431542 significantly reduced lactate generation and the fluorescence intensity of 2-NBDG (Fig. 5C, D). Similarly, western blot analysis showed that TGF-β inhibition by SB-431542 restored the expressions of NDUFB1, UQCRCII, COXIV, and ATP5A, whereas reduced the expressions of HIF-1α, HK2, PKM2, and LDHA under NSG1 overexpression (Fig. 5E), suggesting that TGF-β is indispensable for NSG1-induced metabolic reprogramming in ESCC.

### Metabolic reprogramming induced by NSG1/TGF-β axis results in EMT activation

**Fig. 6:**
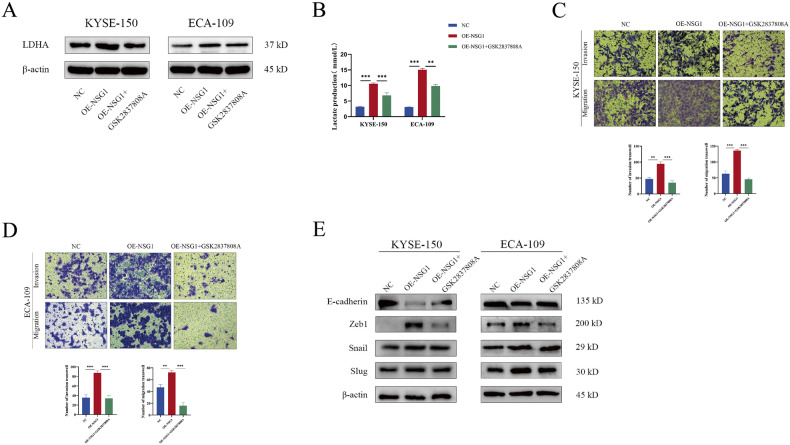
Metabolic reprogramming induced by NSG1/TGF-β axis results in EMT activation. **A**–**E** KYSE-150 and ECA-109 cells were transfected with NSG1 and treated with GSK2837808A (15 µM). **A** Protein levels of LDHA present in ESCC cells were quantified using western blot analysis. **B** Lactate production was determined by enzymatic assays. **C**, **D** The capacities of migration and invasion in ESCC cells were assessed via a transwell assay, 200×. **E** Protein levels of E-cadherin, Zeb1, Snail, and Slug were detected by western blot. Data from three independent experiments are presented the mean ± SD. *P < 0.05, **P < 0.01, and ***P < 0.001.

The lactate content is represented as a crucial component of the Warburg effect and lactate dehydrogenase (LDH), being the chief metabolic enzyme, is tasked with the conversion of pyruvate into lactate [32, 33]. Considering that TGF-β is vital for the regulation of LDHA and tumor migration [34, 35], we deduced that the NSG1/TGF-β axis might induce an EMT-like phenotype in ESCC cells by activating LDHA. Correspondingly, ESCC cells underwent treatment with the LDHA selective inhibitor GSK2837808A (15 µM) for a span of 24 hours aimed at suppressing LDHA expression (Fig. 6A). This treatment resulted in a noticeable drop in both LDHA and extracellular lactate content (Fig. 6B). Further, evidence from the transwell assay suggests that GSK2837808A’s presence significantly reduced NSG1’s potential to encourage migration and invasion within ESCC cells (Fig. 6C, D). Accordingly, GSK2837808A application restored E-cadherin expression and concurrently downregulated Zeb1, Snail, and Slug expression (Fig. 6E). Notably, GSK2837808A had no effect on ESCC cell proliferation, implying that LDHA might not be a significant element of the NSG1/TGF-β axis to stimulate the growth of ESCC cells (Fig. S2A). Together, these findings indicate that LDHA might serve as a downstream effector molecule of the NSG1/TGF-β axis responsible for EMT activation of ESCC cells.

### TGF-β inhibition diminishes NSG1-mediated ESCC growth in xenografted mice

**Fig. 7:**
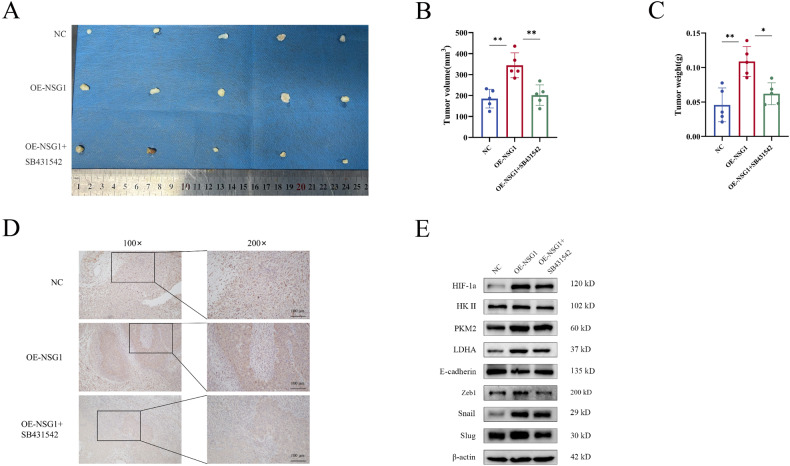
TGF-β inhibition diminishes NSG1-mediated ESCC growth in xenografted mice. **A**–**C** Tumor xenograft experiments were carried out using NSG1 overexpression KYSE-150 cells compared with control cells and NSG1 overexpression cells with SB431542 (5 ml/mg). Tumor volume and weight were analyzed (right). **D** The representative images displayed depict immunohistochemical staining from serial sections of xenograft tumor samples, specifically stained for Ki-67. Scale bars, 100 µm. **E** Protein levels of HIF-1α, HK II, PKM2, LDHA, E-cadherin, Zeb1, Snail and Slug were detected by western blot. Data from three independent experiments are presented the mean ± SD. *P < 0.05, **P < 0.01, and ***P < 0.001.

Finally, to investigate the impact of the NSG1/TGF-β axis on tumor development in vivo, a xenograft model featuring stable NSG1 overexpression was established. The results showed that NSG1 overexpression boosted tumor weight, volume, and Ki-67 expression, while SB-431542 treatment inhibited the ability of NSG1 on tumorigenesis in vivo (Fig. 7A–D), demonstrated that NSG1/TGF-β axis is indispensable signaling for proliferation of ESCC cells. Consistent with expectations, western blot analyses of xenograft tumors validated the impact of NSG1 overexpression on the upregulation of glycolysis-associated and EMT-associated protein expression, which include HIF-1α, HK2, PKM2, LDHA, E-cadherin, Zeb1, Snail, and Slug in vivo. Conversely, the suppression of TGF-β proved capable of reversing this impact (Fig. 7E). These outcomes not only validate the role of the NSG1/TGF-β axis in enhancing tumor glycolysis and promoting ESCC progression, but also offer additional insight into ESCC development.

## Discussion

In this research, we detected an extraordinarily high expression of NSG1 in ESCC tissues. We noted an interaction between NSG1 and TGF-β within ESCC cells, demonstrated by the heightened levels of TGF-β protein in both cell lysate and culture supernatant during NSG1 overexpression. Consequently, we analyzed the effect of the NSG1/TGF-β pathway on ESCC progression. As a neuronal endosome protein, NSG1 is vital for vesicular trafficking in neuronal axons. Recent study revealed that NSG1 might participate in the tumor occurrence and development by influencing the JAK/STAT signaling pathway and cyclin D1 [36], whereas the role of NSG1 on ESCC development remains to be uncovered. Here we discovered that high NSG1 expression existed in both cancer tissues and cancer cell lines. and led to a dismal prognosis for ESCC patients. Additionally, high NSG1 expression was shown to be associated with tumor size, indicating that NSG1 might be responsible for the proliferation of ESCC cells.

Next, we investigated the feasibility of NSG1 as a potential therapeutic target for ESCC. Our findings indicated a positive correlation between NSG1 and TGF-β, a principal growth factor exhibiting high expression in ESCC tissues. The activation of TGF-β signaling has been illustrated to significantly influence tumor development. Specifically, TGF-β can trigger the classic TGF-β/Smad signaling pathway by intensifying Smad2/3 phosphorylation, thereby igniting the EMT process in various malignancies such as esophageal, gastric, and lung cancers [37–40]. Our investigation reveals TGF-β as a downstream target of NSG1, an observation supported by the upregulation of the TGF-β/Smad pathway. Furthermore, a reduction in TGF-β expression instigated a decline in tumor proliferation and EMT processing. In line with these results, in vivo experiments, utilizing the ESCC xenograft model in nude mice, confirmed that suppressing TGF-β led to a decrease in tumor weight, volume, and Ki67 expression.

Acting as a crucial orchestrator of metabolic reprogramming, TGF-β/Smad2/3 signaling has demonstrated involvement in the development of numerous malignancies. The potential influence of NSG1 on the metabolic reprogramming of ESCC cells via TGF-β signaling regulation deserves exploration. Consistent with expectations, ESCC cells with NSG1 overexpression showed an upregulation of the majority of glycolysis-related genes, notably, aberrant expression of HIF-1α [41], and a decrease in mitochondrial respiratory activity. Although NSG1 boosted aerobic glycolysis, the total ATP presented negligible change, which might be associated with the less ability of glycolysis than oxidative phosphorylation in producing ATP [42]. In addition, inhibition of TGF-β markedly reversed the NSG1-driven metabolic reprogramming.

As an emerging hallmark of cancer, metabolic reprogramming has been revealed to exert extensive cross-talk with EMT [43]. This study further demonstrates that EMT activation is facilitated by metabolic reprogramming accompanied by elevated glycolysis levels. TGF-β is reported to stimulate the EMT process, accumulate lactic acid in the tumor microenvironment, which is closely correlated with the spread of malignancies, and then promote the tumor’s spread and proliferation [31]. As one of the critical enzymes that catalyzes the conversion of pyruvate to lactate, LDHA is intimately associated with several malignancies. LDHA can accelerate the growth of ESCC and hasten its metastasis by altering the gene expression of AKT and cyclin D1 [44]. Therefore, whether TGF-β-induced LDHA change has impact on glucose metabolism of ESCC cells is worth exploring. Promisingly, by inhibiting LDHA, acting on the NSG1/TGF-β axis, significant changes were observed which manifested as a notable decrease in the invasion and migratory abilities of ESCC cells. This was also accompanied by diminished expression levels of EMT-related genes including E-Cadherin, Zeb1, Snail, and Slug.

**Fig. 8:**
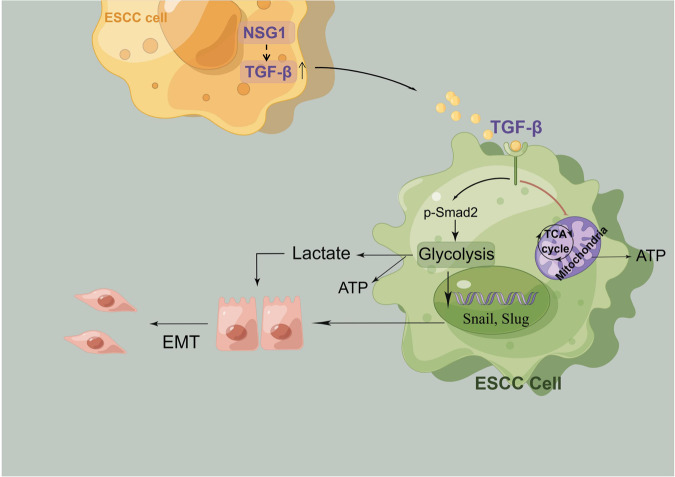
A proposed working model that illustrates how the NSG1/TGF-β axis promotes the development of ESCC. NSG1 promotes glycolytic metabolism via the TGF-β signaling, enhancing the progression of ESCC and EMT.

In conclusion, these findings provide an enhanced understanding of the NSG1/TGF-β axis’s role in ESCC development and propose a potential target for ESCC therapy (Fig. 8). However, it must be acknowledged that the mechanism behind NSG1 expression leading to TGF-β upregulation remains uncertain and warrants further investigation in future studies.

## Materials and methods

### Patients and specimens

This investigation incorporated 45 ESCC samples, complimented with their matched non-cancerous specimens, all of which were sourced from Fujian Provincial Hospital. Furthermore, each specimen underwent a verified pathological diagnosis.

### Cell culture and lentivirus transfection

The Chinese Academy of Sciences (Shanghai, China) provided the human ESCC cell lines KYSE150, TE-1, and HEEC, while ECA109 was sourced from Pronox Life Sciences Company (Wuhan, China). The cell culture process involved using RPMI 1640 medium (HyClone, USA), enriched with 10% fetal bovine serum (FBS) (Procell, China). After preparation, all cell lines were maintained at 37 °C in a cell culture incubator with a controlled environment of 5% CO_2_.

Plasmids designed for lentivirus were acquired from Genechem Co., Ltd (Shanghai, China). To ensure optimal viral infection, a specific multiplicity of infection (MOI) value of 10 was employed when adding the lentivirus to the cell culture plate. Following a 72-h incubation period, puromycin (Sigma-Aldrich, USA) was introduced to facilitate the selection of ESCC cell lines that exhibited stable gene expression. Subsequently, qPCR and western blot analyses were conducted to assess the transfection efficiency.

### Immunohistochemistry

Paraffin-embedded tissue sections were de-paraffined and re-hydrated. Microwaves were used to retrieve antigens. The tissues were blocked with 0.3% hydrogen peroxide solution for a duration of 10 minutes, followed by 1% goat serum. The primary antibody NSG1 (1:100, PA5-36497, Invitrogen) or Ki-67 (1:500, # 12202 S, Cell Signaling Technology) was added dropwise to the tissues and incubated overnight at a temperature of 4 °C. The tissue samples were subsequently incubated with biotinylated secondary antibodies at room temperature for a duration of 10 minutes. Following incubation, the samples were stained using diaminobenzidine (DAB) and hematoxylin. Each staining was independently assessed by two pathologists; moreover, throughout the experiment, the investigators were kept blinded to the allocation of groups.

### Quantitative Real-time PCR analysis

Total RNA was isolated from ESCC cells using TRIzol (Macherey-Nagel, Germany). After extraction, cDNA synthesis followed by qRT-PCR was performed as per the GoScript™ Reverse Transcriptase Mix and GoTaq® qPCR Master Mix (Promega, USA) protocols respectively. Table S2 details the sequences of the utilized primers.

### RNA sequencing

Briefly, total RNA was extracted from NSG1 overexpressing and negative control cells using the NucleoZOL reagent. Each sample was prepared in triplicate and RNA sequencing and analyses were performed. RNA sequencing was executed using the Illumina platform (Shanghai Genechem Co., Ltd.). The subsequent analysis of the RNA sequencing data was performed using the DESeq2 and ClusterProfiler software packages.

### Bioinformatic analysis

Data pertaining to the expression and survival of NSG1 and TGF-β were obtained from the CBio Cancer Genomics Portal for patients diagnosed with ESCC (https://www.cbioportal.org/).

### Immunofluorescence

The subcellular localization of proteins was investigated with ESCC cells adhered to coverslips. These cells were fixed for 15 min using 4% paraformaldehyde, followed by a rinse. Subsequently, the cells were permeabilized for 15 min with 0.2% Triton X-100 (Solarbio, China). After that, 5% goat serum was applied for 15 min as a blocking treatment. The cells were subsequently incubated with primary antibodies NSG1 (1:200, PA5-101719, Invitrogen) and TGF-β (1:100, MA1-21595, Invitrogen) overnight at 4 °C. After PBS rinses, the cells were subjected to staining with a secondary antibody (goat anti-rabbit Alexa Fluor 647, 1:500, ab150079, Abcam; goat anti-mouse Alexa Fluor 488, 1:500, ab150113, Abcam) for 1 h at room temperature. In conclusion, the cells were stained with an anti-fading fluorescent solution containing DAPI, and were then observed under laser scanning confocal microscopy.

### Western blot analysis

Total protein was extracted from both ESCC cells and the cancerous tissue of ESCC patients utilizing the protocol of the complete protein extraction kit (Solarbio, China). After extraction, the protein was subjected to SDS-PAGE electrophoresis. This was followed by transferring the proteins onto a PVDF membrane (Millipore, USA), which was then blocked with a 5% skim milk solution in Tris-buffered saline/Tween (TBST). A primary antibody was then incubated overnight at 4 °C on the membrane. The following day, the PVDF membrane was cleansed with TBST (Beyotime, China) before it was incubated with goat anti-rabbit IgG (1:50000, HA1001, HUABIO) for 1 h at room temperature. Finally, the visibility of the proteins was enhanced using the right developing solution (Thermo Fisher Scientific, USA). Details about the utilized antibodies are available in the Supplementary Table 3.

### Coimmunoprecipitation

To validate the interaction between NSG1 and TGF-β, the overexpressed Flag-NSG1 plasmid was transfected into KYSE-150 and ECA-109 cells. Whole-cell lysates were subsequently incubated with antibodies against Flag and TGF-β, or with IgG antibodies, at 4 °C overnight. The immune complex solution is combined with protein A/G magnetic beads, followed by an hour-long incubation at room temperature. Subsequently, a washing step is performed to eliminate any unbound immune complexes. To conduct western blot analysis, 100 μl of sample loading buffer was added to the sample, which was then placed in a metal bath and heated at a temperature of 96–100 °C for a duration of 10 minutes. This procedure was performed in order to effectively separate the supernatant, which contains the desired antigen (Pierce™ Classic Magnetic IP/Co-IP Kit, America).

### Enzyme-linked immunosorbent assay (ELISA)

Human TGF-β ELISA kits were purchased from Abcam (ab100647) and carried out the assays following the manufacturer’s instructions. The supernatant, harvested from cells incubated in 6-well plates over 24 h, was uniformly blended with a buffer and a color developer. This blend was incubated at room temperature under dark conditions with mild stirring for 30 minutes. After adding the stop solution, we immediately recorded the absorbance at 450 nm.

### RNA Interference

Lipofectamine^TM^ 3000 (Invitrogen, USA) was used to introduce small interfering RNA (siRNA) duplexes into cells according to the manufacturer’s instruction. Following a 48-hour incubation period, the cells were harvested for subsequent experiments. The siRNA products used in the experiments were procured from Zolgene Biotechnology Co., Ltd (Fuzhou, China). The siRNA sequences were as follows: GGUGAAGAAGCUAAAGAAATT, and the antisense was as follows: UUUCUUUAGCUUCUUCACCTT.

### CCK-8 assays

A total of 2 × 10^3^ ESCC cells were singly allocated into each well of a 96-well plate, subjected to a minimum of three replicates per category, and were incubated for 24 h. Staggered through various intervals (0 h, 24 h, 36 h, 48 h, and 72 h), 10 μl of CCK-8 reagents (Beyotime Biotechnology, China) were introduced to these cells which were then incubated at 37 °C with 5% CO_2_ for 2 h. Finally, the microplate reader documented the readings at 450 nm.

### Cell clone formation experiment

A total of 1 × 10^3^ ESCC cells were individually seeded into the wells of a 6-well plate, then incubated for a period of 2 weeks. Post-incubation, the cells were subjected to crystal violet staining for enhanced visualization. Subsequently, the colonies were enumerated using a light microscope.

### Transwell assays

Cells approximately numbering between 2 × 10^4^ and 5 × 10^4^ were inoculated in the upper section of a 24-well transwell chamber (Coring, USA), which featured a pore size of 8 μm. For invasion assays, cells were seeded in the upper chambers coated with Matrigel (BD Biosciences, USA). Following that, RPMI 1640 medium, supplemented with 10% FBS, was introduced into the bottom chamber. After two days, the cells that had adhered to the bottom surface of the transwell membrane were stained with crystal violet. Subsequently, they were observed and quantified under a light microscope.

### Seahorse experiment

The Seahorse XF24 flux analyzer (Seahorse Bioscience, America) was utilized to measure extracellular acidification rates (ECAR) and real-time ATP levels. The cells were seeded 24 h prior to the experiment. Cells were seeded a day before the experiment and cultured at a concentration of 1 × 10^5^ cells per well in the Seahorse XF cell culture plate with an overnight incubation period. Prior to analysis, the culture medium was changed into 500 μL assay medium, and cells were incubated in a 37 °C non‐CO_2_ incubator for 1 hour. The glycolysis pressure test injects 10 mM glucose (56 μL), 1.0 μM oligomycin (62 μL), and 50 mM 2-deoxyglucose (2-DG, 69 μL) into the respective injection port of the sensor cartridge. Real-time ATP rates inject 1.5 μM oligomycin (56 μL) and 0.5 μM rotenone + Antimicrobial A (Rot/AA,62 μL) into the respective injection port of the sensor cartridge. Then the result was automatically calculated by the Seahorse XF-24 analyzer.

### Lactic acid production assays

The lactic acid analysis was conducted in accordance with the Lactic Acid Assay Kit guidelines (Nanjing Jiancheng Corporation, China). Cell supernatant, cultivated in 12-well plates for 24 h, was collected and thoroughly mixed with buffer and color developer. The mixture then underwent a reaction in a water bath set at 37 °C for a duration of 30 min. Subsequently, the activity of LDH was quantified at 570 nm using a microplate reader.

### 2-NBDG uptake assays

The cellular capacity for glucose uptake was assessed utilizing the fluorescent glucose analog, 2-NBDG (APExBIO, America). Cells, positioned in 6-well plates, were grown in glucose-free DMEM medium for a period of 6 hours. Following three sequential PBS washes, cells were given 50 μM 2-NBDG and allowed to incubate for half an hour. Imaging was performed in five randomly chosen fields of view under a fluorescence microscope.

### Animal experiments

Xenografts were established via a subcutaneous injection of a 100 µL solution containing 1 × 10^7^ KYSE-150 cells, administered to null mice. The mice were then randomly divided into three groups (*n* = 5): the vehicle-only group, the NSG1-OE group, and the NSG1-OE + SB-431542 (5 mg/kg) group. Three weeks following tumor implantation, the mice were euthanized for subsequent tumor analysis. The dimensions of the tumor were assessed every two days with a digital caliper, and its volume was calculated according to the formula: length × breadth ^2^ × 0.5.

### Ethics statement

The research involving human subjects was meticulously reviewed and received approval from the Ethics Committee of Fujian Provincial Hospital (approval number: K2019-12-038). Each participant provided written informed consent for their involvement in this study. The Animal Ethics Committee of Fujian Ambri Biotechnology Co., Ltd. accorded approval to all animal experiments conducted in this study (approval number: IACUC FJABR 2022121501).

### Statistics analysis

Means and standard deviations (SD) are represented in the data. T-tests were utilized to evaluate the differences between two groups, whereas for evaluations across multiple groups, one-way analysis of variance (ANOVA) was employed. Count data comparisons were undertaken using the χ2 test. The significance of a study is established when the *p*-value is less than 0.05. All statistical analyses were administered using SPSS 26.0 (IBM Corp., USA).

### Supplementary information

Supplementary Figure 1.

Supplementary Table

Supplementary Figure 2.

Supplementary Figure legend

Original Data File

## Data Availability

All data pertinent to this study are contained within the article or can be obtained from the corresponding author upon a reasonable request.
