# Supplementary figures and images for: NSG1 promotes glycolytic metabolism to enhance Esophageal squamous cell carcinoma EMT process by upregulating TGF-β
Source: Cell Death Discov. 2023 Oct 23;9:391. doi: 10.1038/s41420-023-01694-6 (PMC10593808; doi:10.1038/s41420-023-01694-6)

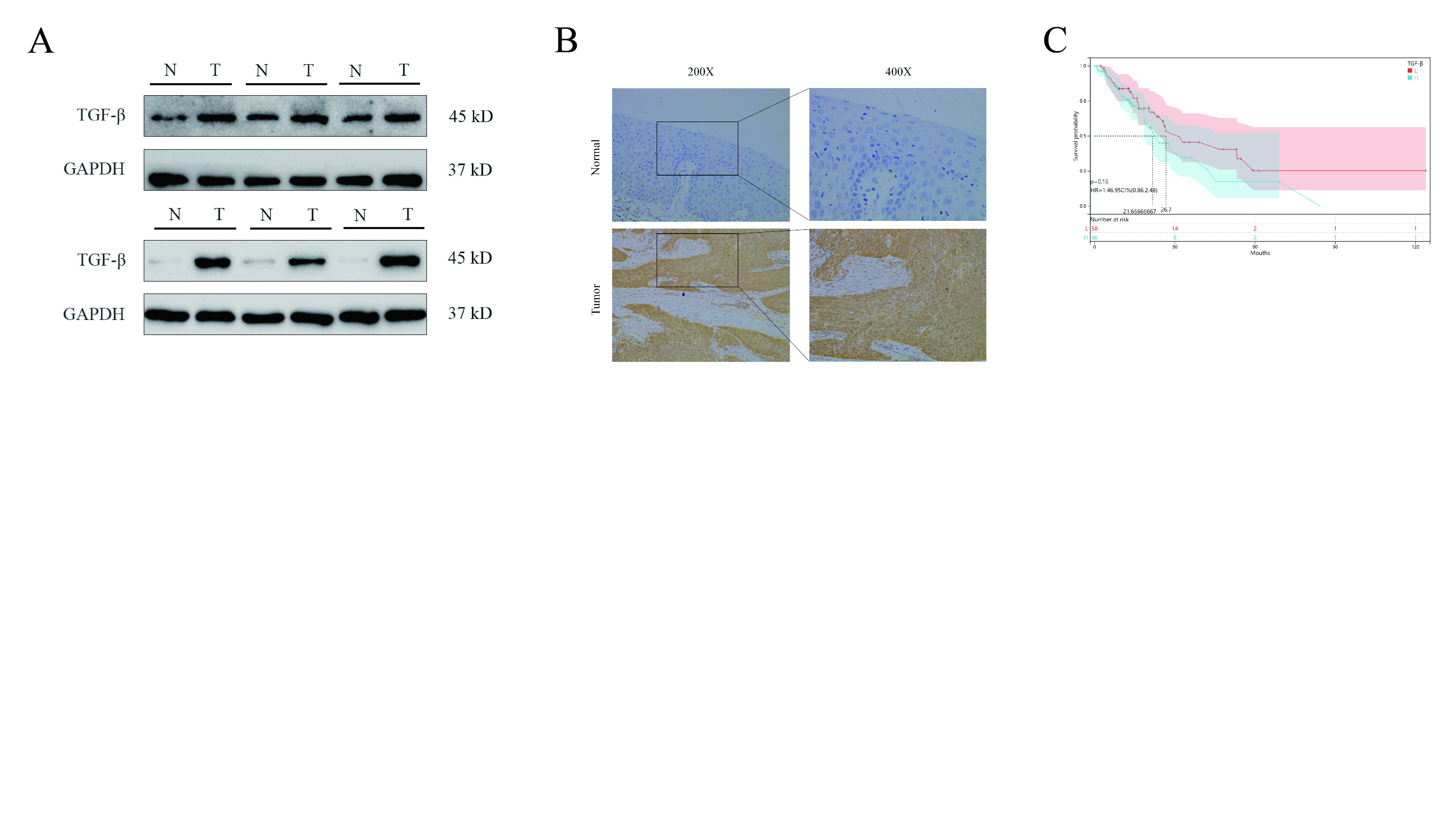

Supplement: Supplementary file 1 — Supplementary Figure 1. [file 41420_2023_1694_MOESM1_ESM.jpg]

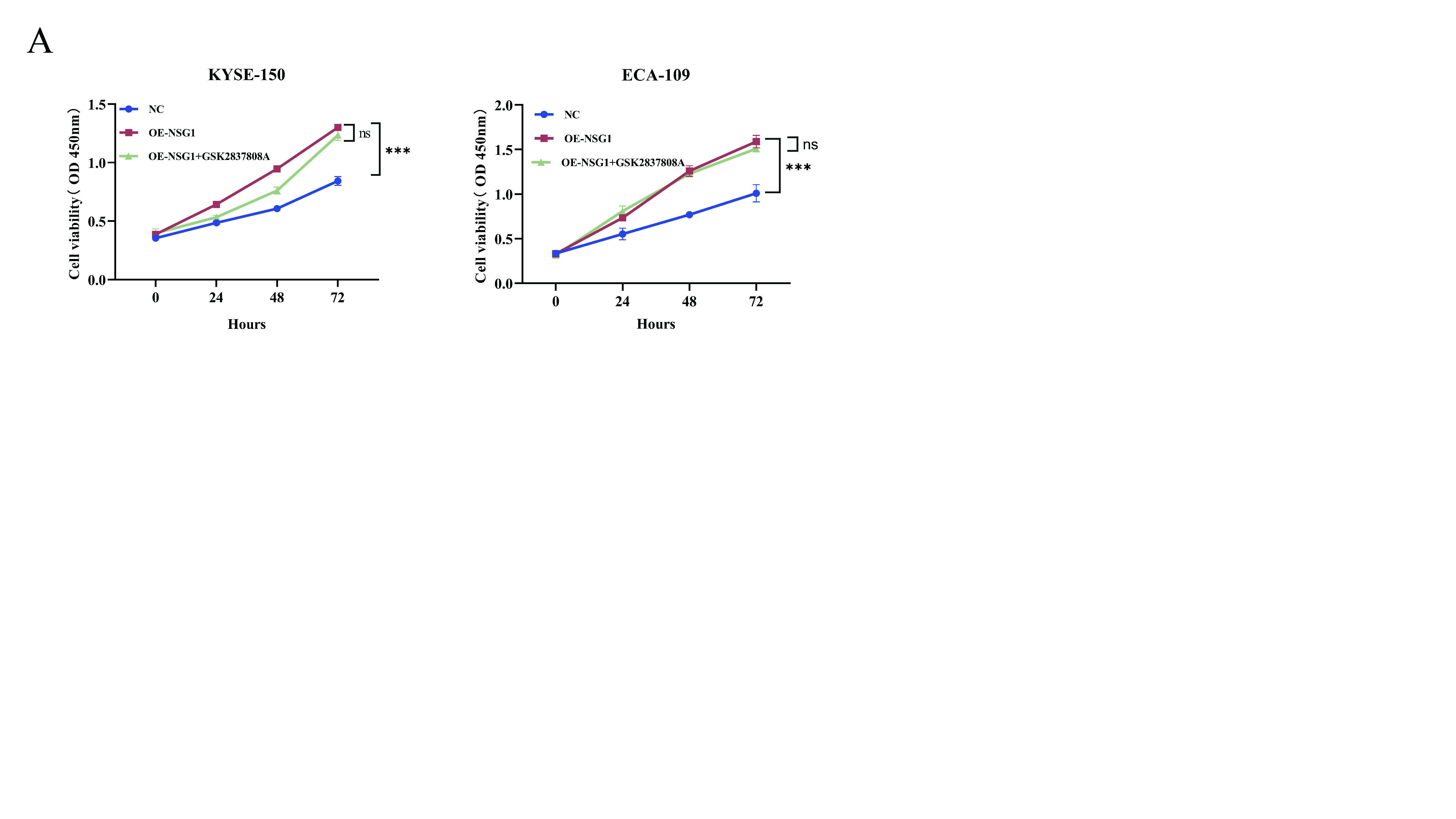

Supplement: Supplementary file 3 — Supplementary Figure 2. [file 41420_2023_1694_MOESM3_ESM.jpg]
