# Supplementary material for: NSG1 promotes glycolytic metabolism to enhance Esophageal squamous cell carcinoma EMT process by upregulating TGF-β
Source: Cell Death Discov. 2023 Oct 23;9:391. doi: 10.1038/s41420-023-01694-6 (PMC10593808; doi:10.1038/s41420-023-01694-6)
Supplement: Supplementary file 2 — Supplementary Table [file 41420_2023_1694_MOESM2_ESM.docx]

**Supplementary Table 1.** Relationship between TGF-β protein expression and clinicopathologic characteristics

| Parameter | Total | TGF-β | | | | P-value |
| --- | --- | --- | --- | --- | --- | --- |
|  |  | High expression | | Low expression | |  |
| Age(y) | | | | | | |
| <60 | 84 | 46 | | 38 | | 0.263 |
| ≥60 | 99 | 46 | | 53 | |  |
| Gender | | | | | | |
| Male | 156 | 79 | | 77 | | 0.68 |
| Female | 28 | 13 | | 15 | |  |
| Histological type | | | | | | |
| ESCC | 96 | 56 | | 40 | | 0.022 |
| ESCA | 87 | 36 | | 51 | |  |
| T classification | | | | | | |
| T1-2 | 75 | 31 | | 44 |  | 0.043 |
| T3-4 | 91 | 52 |  | 39 |  |  |
| N classification | | | | | | |
| N0 | 77 | 39 |  | 38 |  | 0.876 |
| N1-3 | 87 | 43 |  | 44 |  |  |
| M classification | | | | | | |
| M0 | 135 | 71 |  | 64 |  | 0.187 |
| M1 | 26 | 10 |  | 16 |  |  |
| Stage |  |  |  |  |  |  |
| Ⅰ-Ⅱ | 97 | 44 |  | 53 |  | 0.141 |
| Ⅲ-Ⅳ | 61 | 35 |  | 26 |  |  |

**Supplementary Table 2. Primers sequence**

| **Name** | **Primer Sequence (5’-3’)** |
| --- | --- |
| TGF-β-F | GAGCCCTGGACACCAACTAT |
| TGF-β-R | AAGTTGGCATGGTAGCCCTT |
| SCL4A8-F | GGACCACCTTAGCCTCTATTACT |
| SCL4A8-R | CTTCTGCTGTCACGATGTTGT |
| CCND2-F | GCTGTCTCTGATCCGCAAGC |
| CCND2-R | CTCAGTCAGGGCATCACAAGT |
| ZNF264-F | GAAGACCTCTCCCAAGACACCTG |
| ZNF264-R | CCTGAAGTGAGATTCCCTCTGAC |
| RC3H1-F | GCAGTGGATCTCCCTAGCAG |
| RC3H1-R | AGGAACAGATTCTAGCAGGTCAG |
| NSG1-F | AAGGTCTCCGTGTTGGTCCT |
| NSG1-R | GCGTAGTAGCTCTCCAAGCC |
| β-actin-F | TGACGTGGACATCCGCAAAG |
| β-actin-R | CTGGAAGGTGGACAGCGAGG |

**Supplemental Table 3. Antibodies information used for western blot and coimmunoprecipitation**

| **Antibody name** | **Dilution** | **Supplier** | **Cat. No** |
| --- | --- | --- | --- |
| NSG1 | 1:1000 | Invitrogen | PA5-36497 |
| TGF-β | 1:1000 | Cell Signaling Technology | # 3711S |
| p-Smad2 | 1:1000 | Cell Signaling Technology | #18338 |
| Smad2/3 | 1:1000 | Cell Signaling Technology | #8685 |
| E-cadherin | 1:1000 | Cell Signaling Technology | #3195 |
| Zeb1 | 1:500 | HUABIO | HA721318 |
| Snail | 1:1000 | Cell Signaling Technology | #3879 |
| Slug | 1:1000 | Cell Signaling Technology | #9585 |
| HIF-1α | 1:1000 | Cell Signaling Technology | #36169 |
| HKII | 1:1000 | HUABIO | HA500186 |
| PKM2 | 1:1000 | ABclonal | A20991 |
| LDHA | 1:1000 | HUABIO | ER00702 |
| NDUFA13 | 1;1000 | ABclonal | A3782 |
| SDHA | 1:1000 | Cell Signaling Technology | #11998 |
| UQCRC2 | 1:1000 | ABclonal | A4181 |
| COX IV | 1:1000 | Cell Signaling Technology | #4850 |
| ATP5A1 | 1:1000 | Cell Signaling Technology | #18023S |
| β-actin | 1:10000 | HUABIO | R1207-1 |
| GAPDH | 1:1000 | Cell Signaling Technology | #5174 |
| Flag | 1:50 | Cell Signaling Technology | #14793 |
| TGF-β | 1:50 | Biorbyt | orb214661 |
| IgG | 1:500 | Abcam | ab205718 |
