# Supplementary material for: NSG1 promotes glycolytic metabolism to enhance Esophageal squamous cell carcinoma EMT process by upregulating TGF-β
Source: Cell Death Discov. 2023 Oct 23;9:391. doi: 10.1038/s41420-023-01694-6 (PMC10593808; doi:10.1038/s41420-023-01694-6)
Supplement: Supplementary file 4 — Supplementary Figure legend [file 41420_2023_1694_MOESM4_ESM.docx]

**Supplementary Figure 1. TGF-β is upregulated in ESCC.**

**A** The protein expression levels of TGF-β in both ESCC tumor (T) and the adjoining normal tissues (N) were quantified via western blotting. **B** ESCC and adjacent non-tumor tissues were stained using immunohistochemically for TGF-β expression. **C** Kaplan–Meier plots of the correlations between TGF-β expression and ESCC patients' overall survival.

**Supplementary Figure 2. Proliferative capacity of ESCC cells.**

**A** The proliferative ability of ESCC cells, in the presence of GSK2837808A (15µM), was measured using CCK-8 assays. **P* < 0.05, ***P* < 0.01, and ****P* < 0.001
