# Supplementary material for: NSG1 promotes glycolytic metabolism to enhance Esophageal squamous cell carcinoma EMT process by upregulating TGF-β
Source: Cell Death Discov. 2023 Oct 23;9:391. doi: 10.1038/s41420-023-01694-6 (PMC10593808; doi:10.1038/s41420-023-01694-6)

Figure 1. B

NSG1

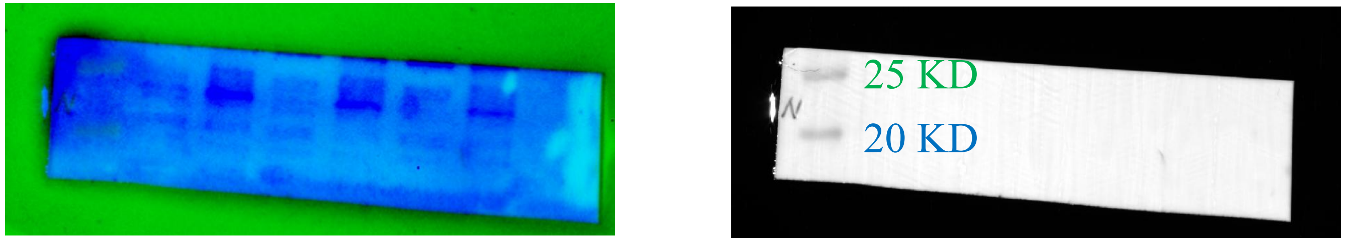

β-actin

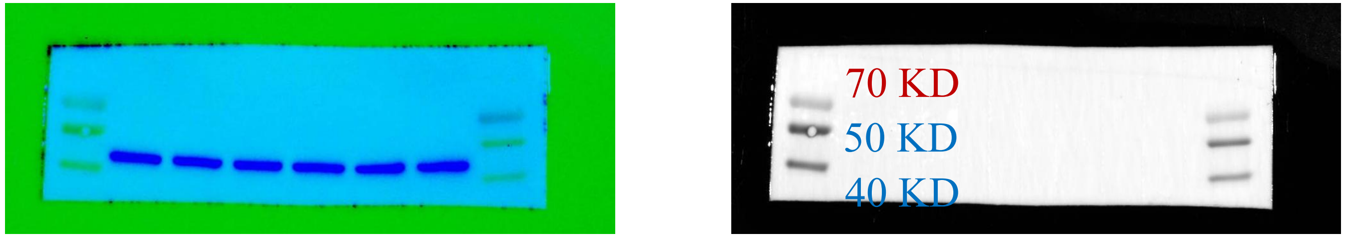

NSG1

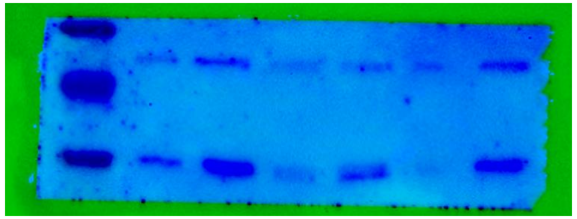

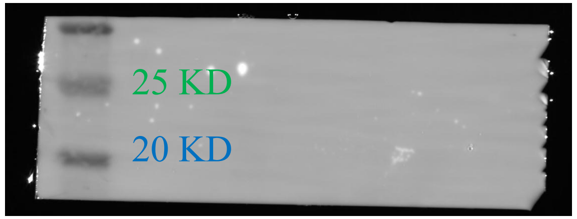

β-actin

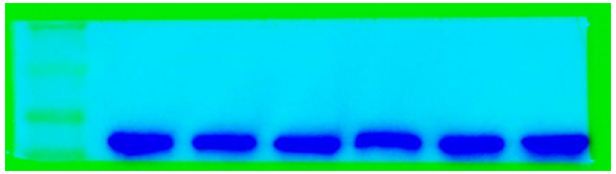

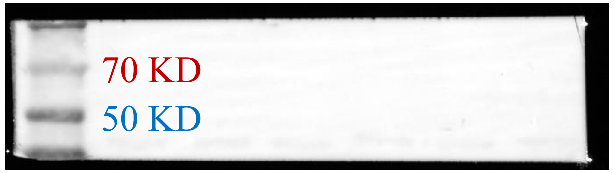

Figure 1. D

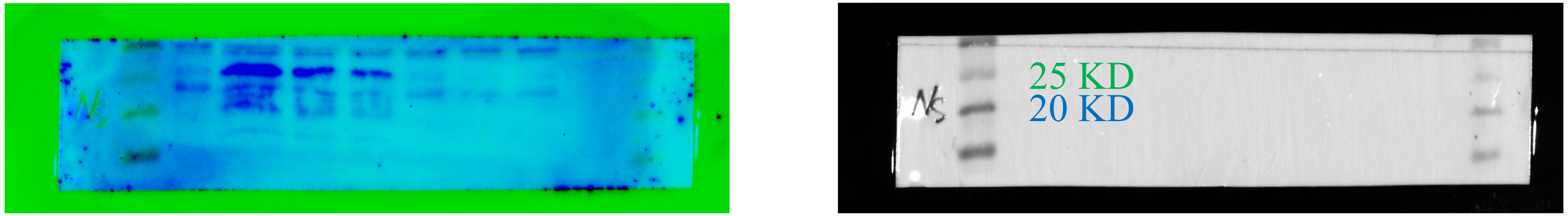

β-actin

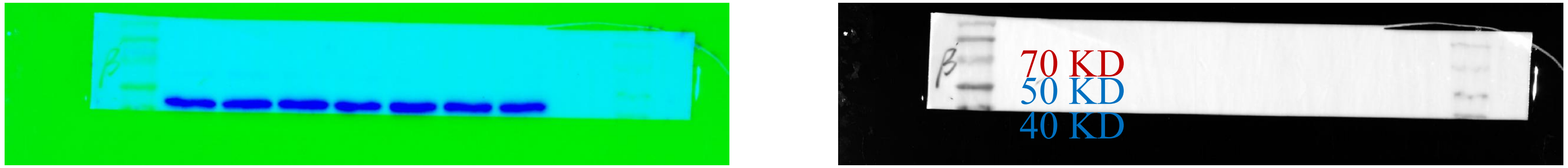

Figure 2. A

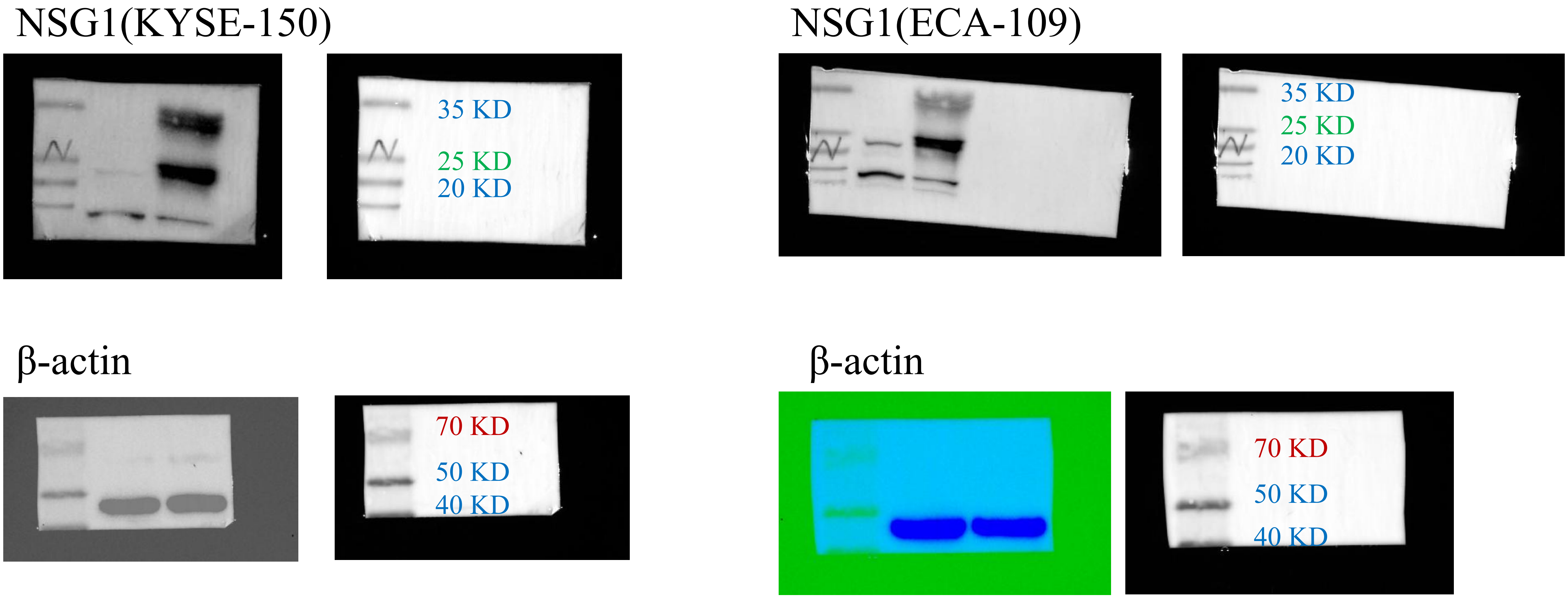
NSG1(KYSE-150)
35 KD
25 KD
20 KD
NSG1(ECA-109)
35 KD
25 KD
20 KD
β-actin
70 KD
50 KD
40 KD
β-actin
70 KD
50 KD
40 KD

Figure 2. E

NSG1

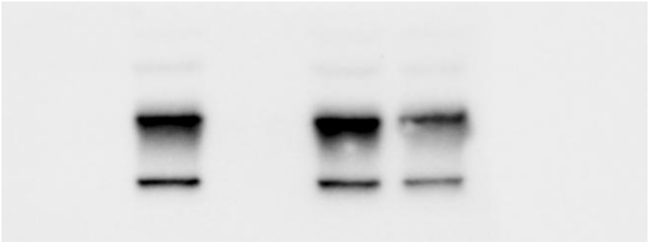

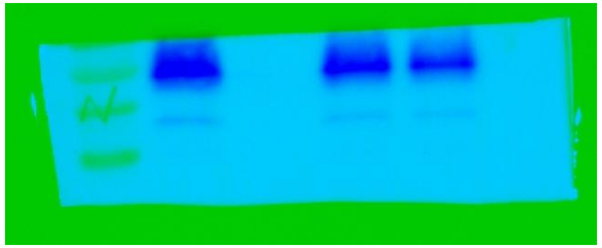

TGF-β

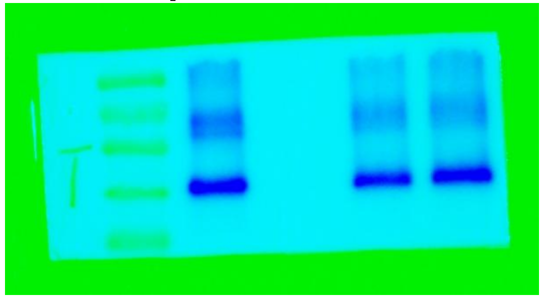

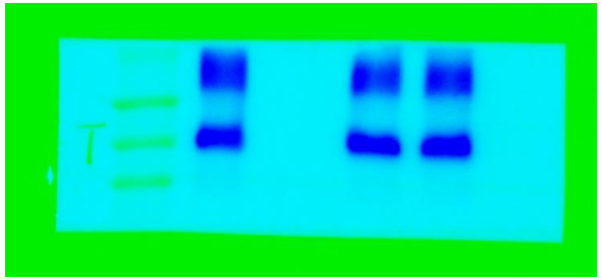

Figure 3. **C**

TGF-β(45 KD)

KYSE-150

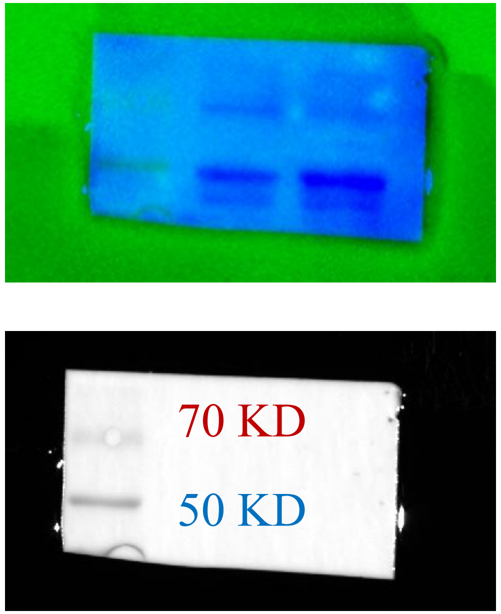

ECA-109

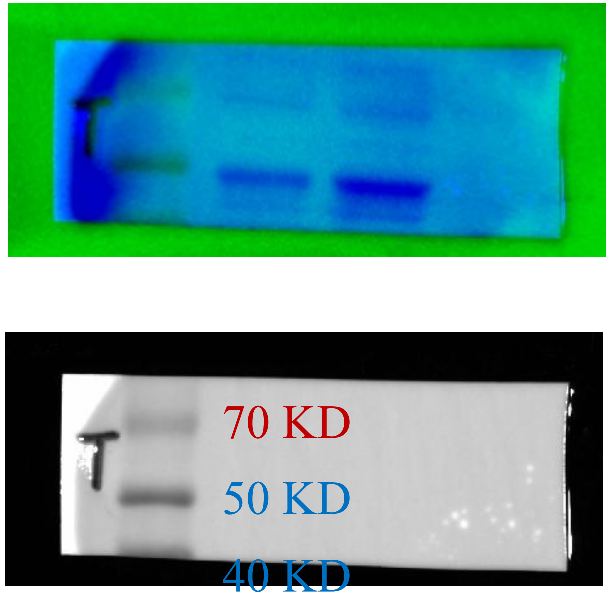

Smad2/3(60,52KD)

KYSE-150

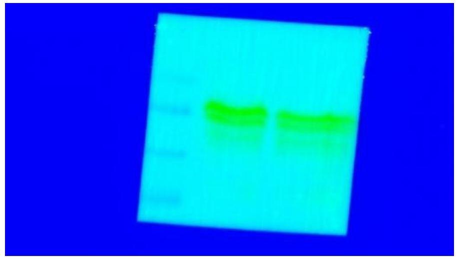

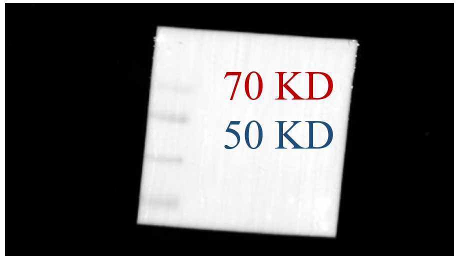

ECA-109

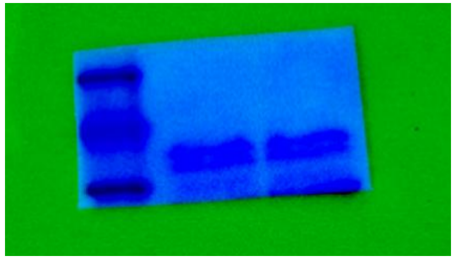

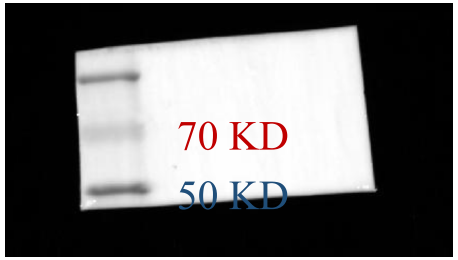

p-Smad2 (60 KD)

KYSE-150

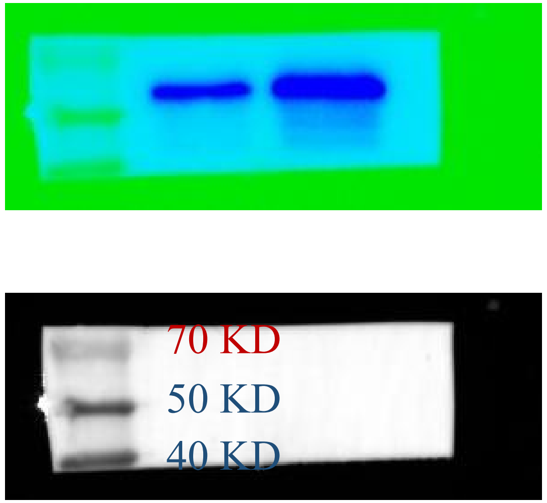

ECA-109

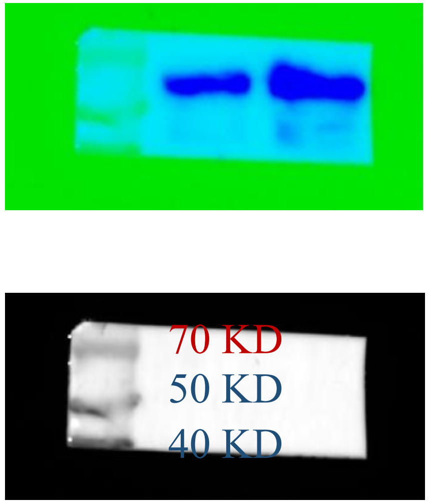

E-cadherin(135 KD)

KYSE-150

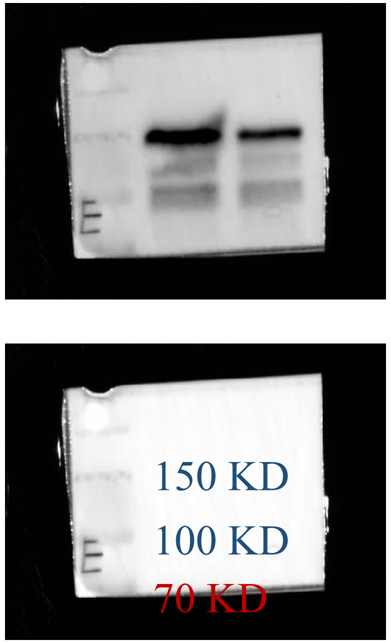

ECA-109

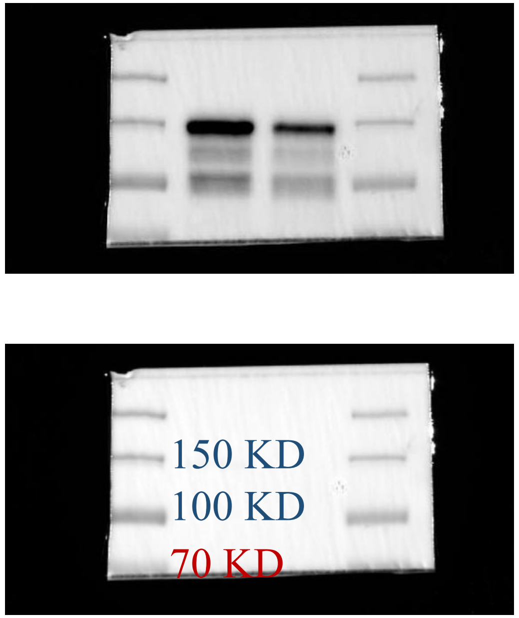

Zeb1(200 KD)

KYSE-150

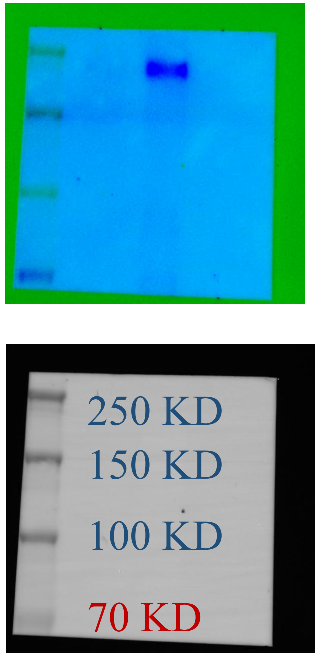

ECA-109

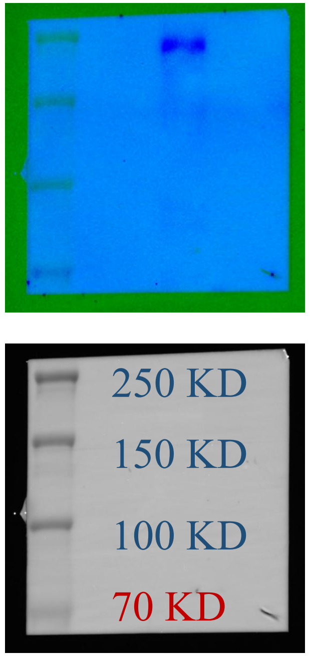

Snail(29 KD)

KYSE-150

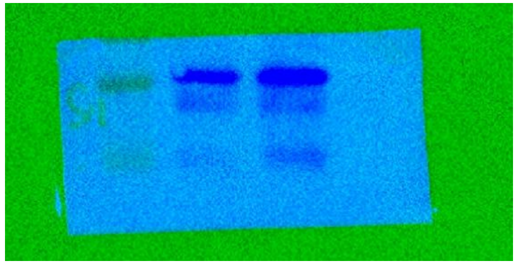

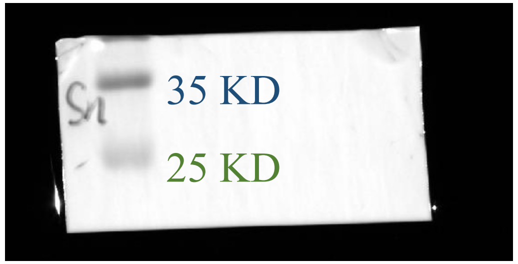

ECA-109

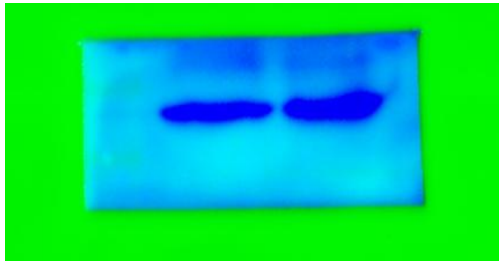

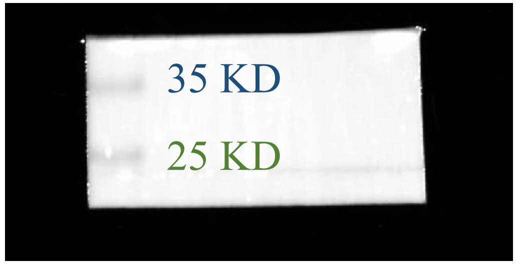

Slug(30 KD)

KYSE-150

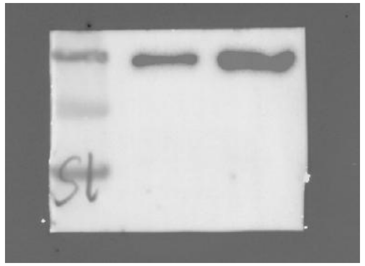

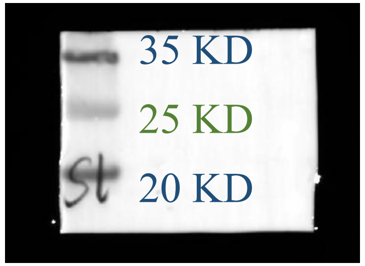

ECA-109

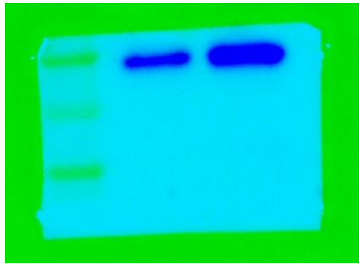

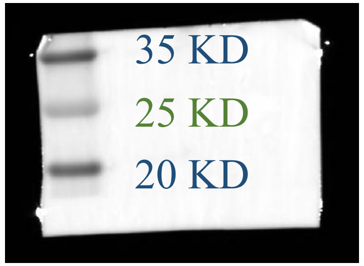

GAPDH(37 KD)

KYSE-150

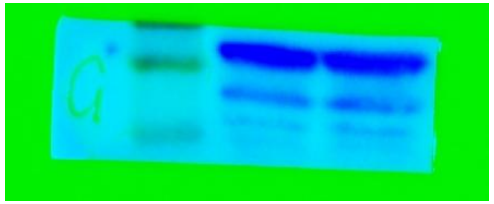

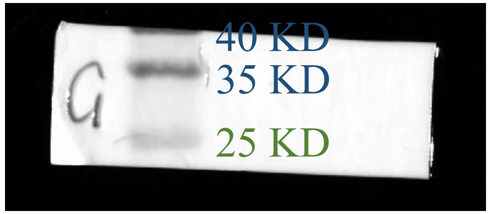

ECA-109

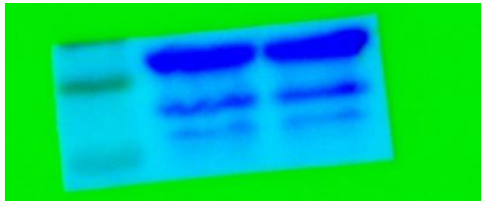

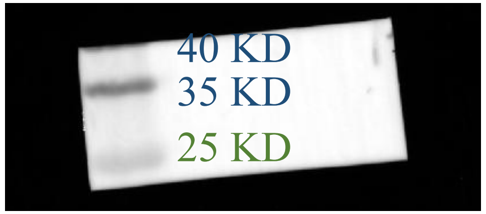

Figure 3. **D**

TE-1

NSG1(21 KD)

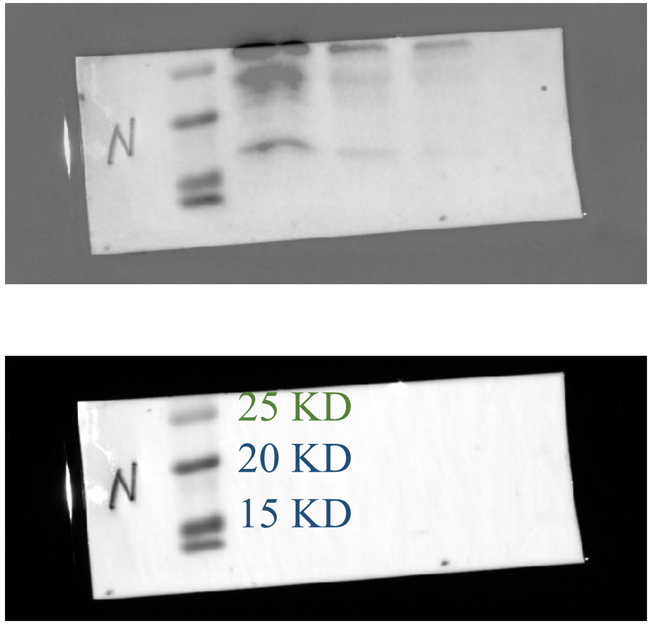

TGF-β(45 KD)

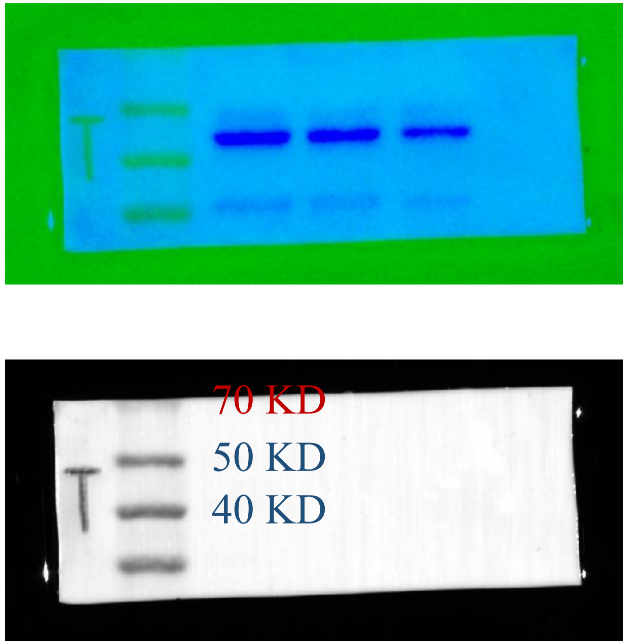

TE-1

Smad2/3(60, 52 KD)

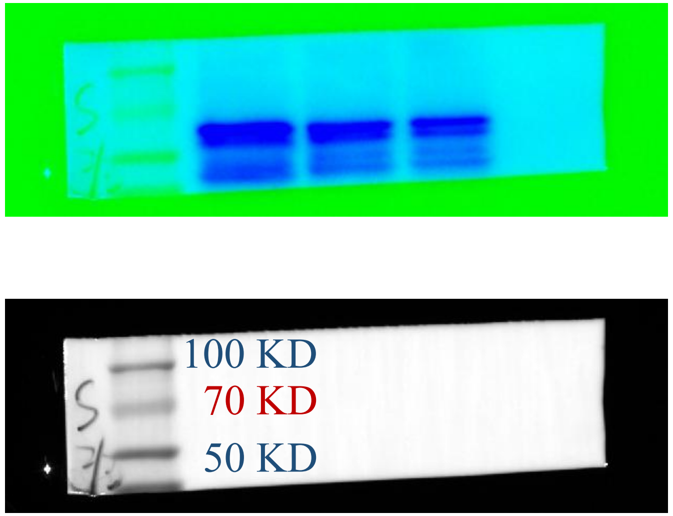

P-Smad2(60 KD)

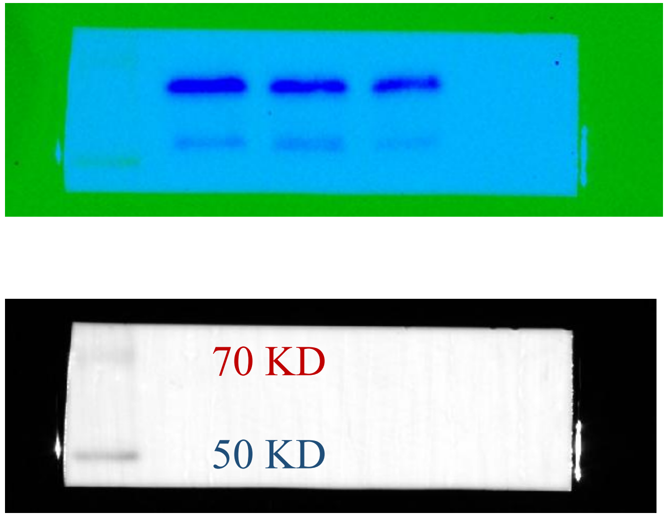

TE-1

E-cadherin(135 KD)

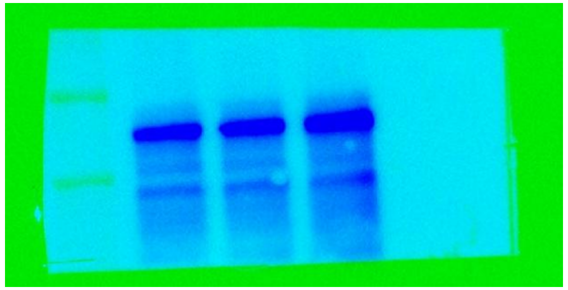

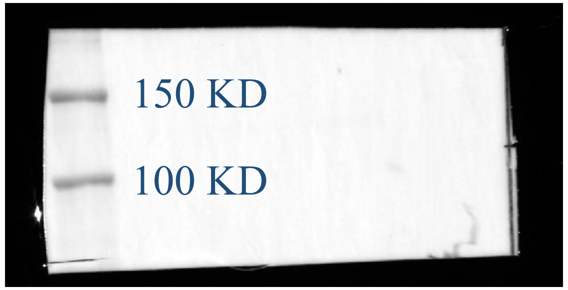

Snail(29 KD)

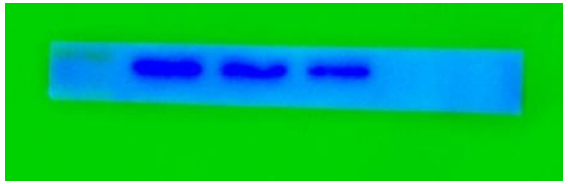

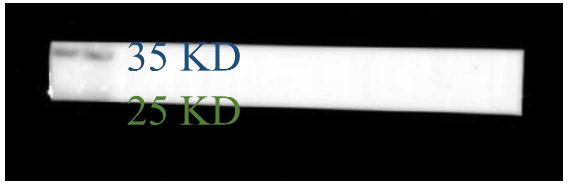

TE-1

E-cadherin(135 KD)

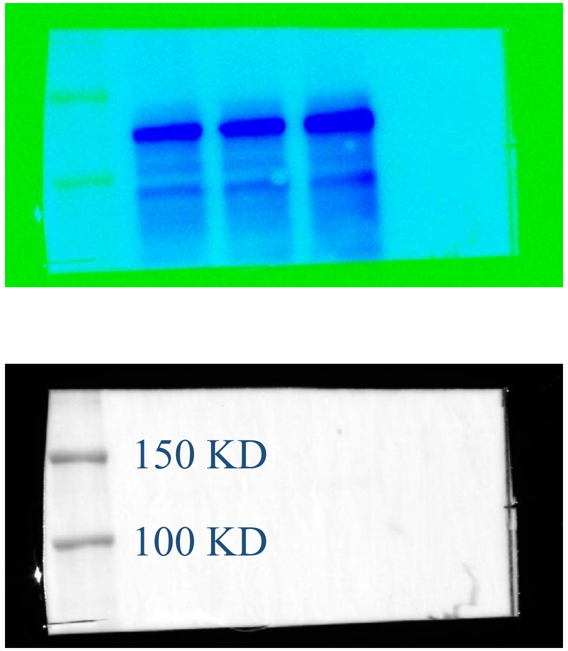

Zeb1(200 KD)

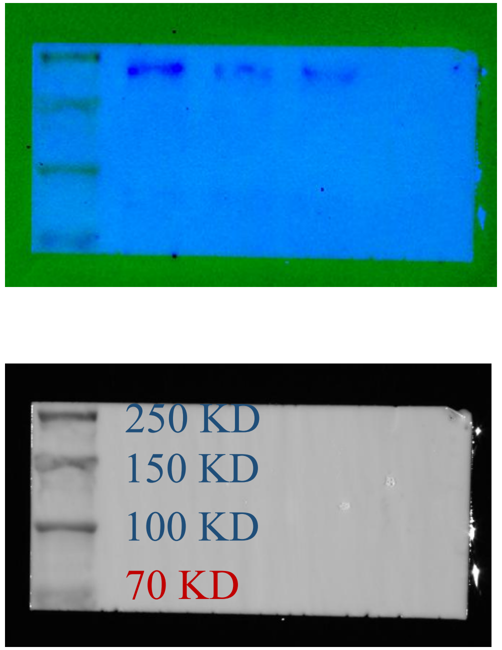

TE-1

Snail(29 KD) Slug(30 KD) GAPDH (37 KD)

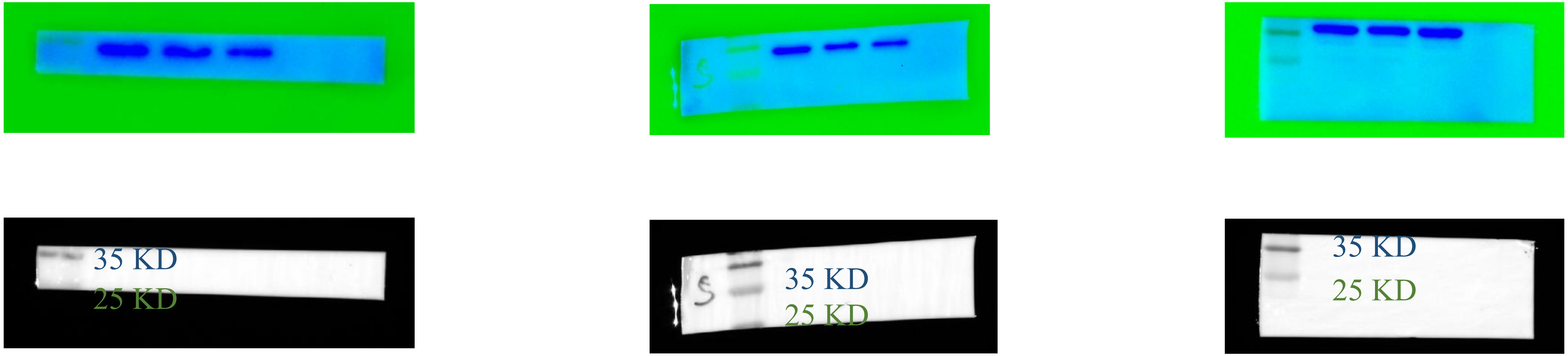

Figure 3. **G**

Smad2/3(60,52 KD)

KYSE-150

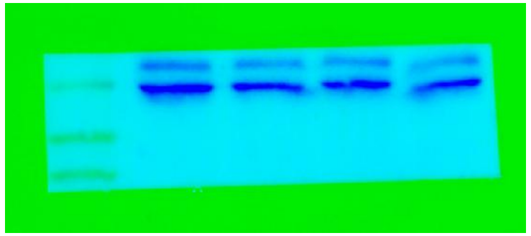

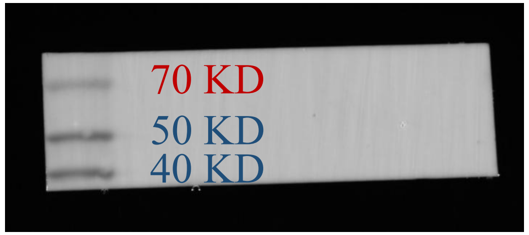

ECA-109

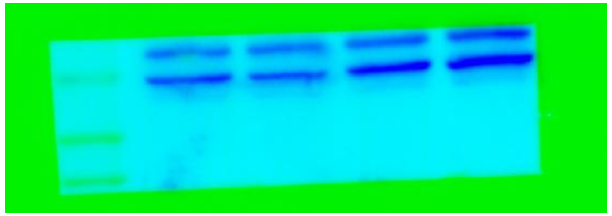

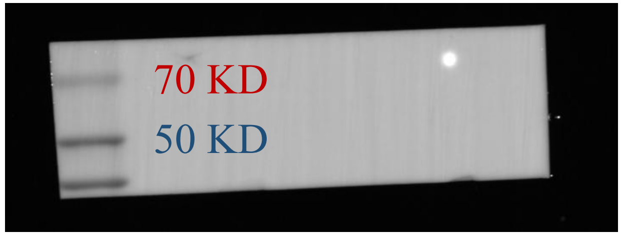

p-Smad2(60 KD)

KYSE-150

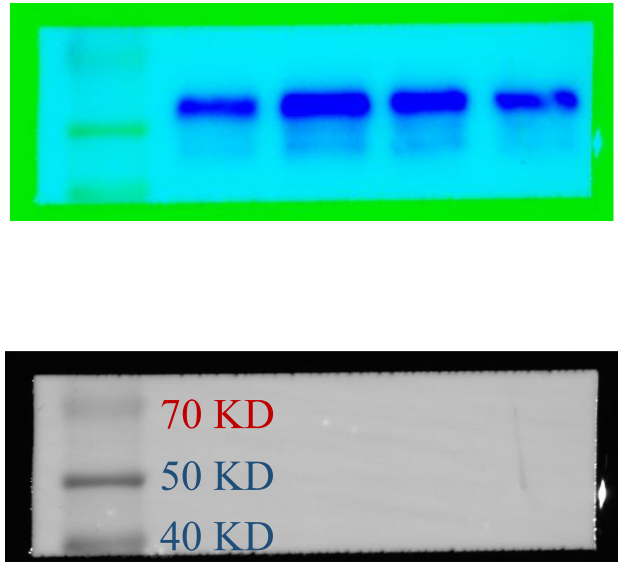

ECA-109

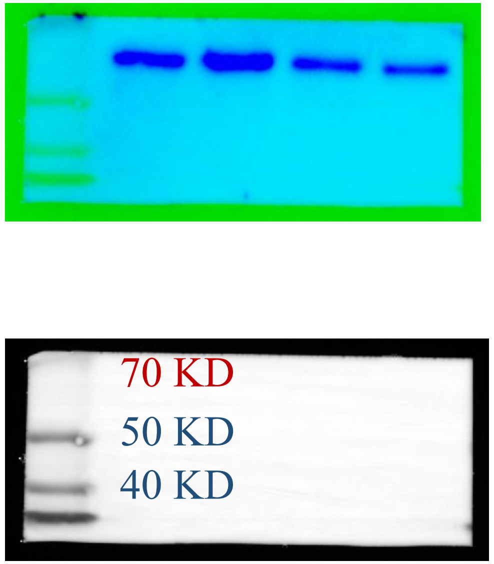

E-cadherin(135 KD)

KYSE-150

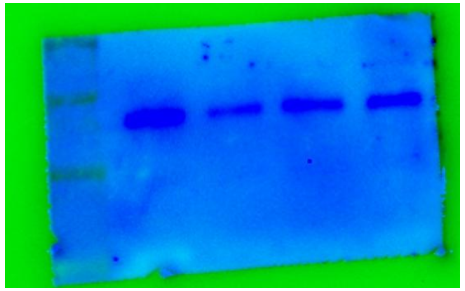

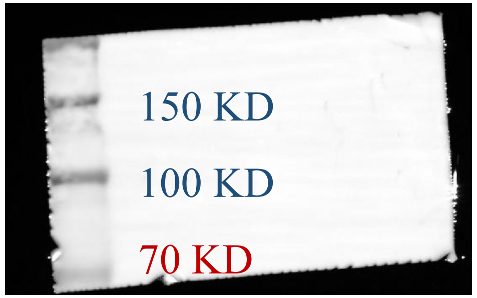

ECA-109

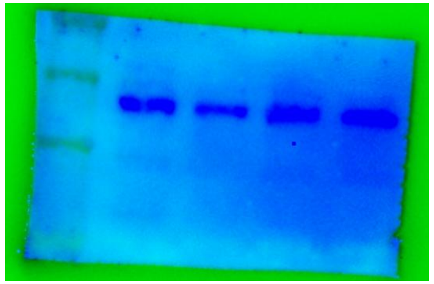

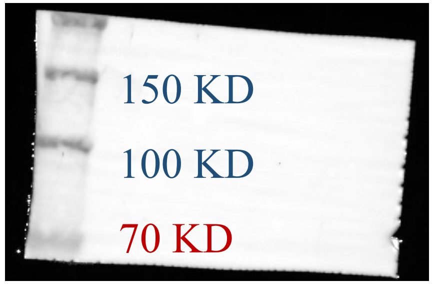

Zeb1(200 KD)

KYSE-150

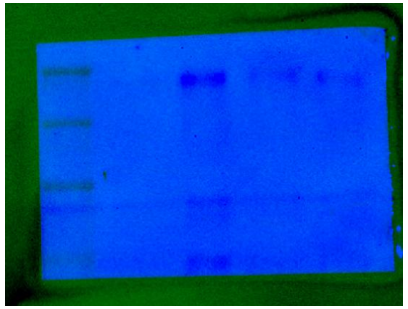

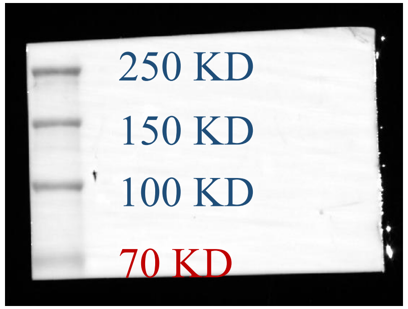

ECA-109

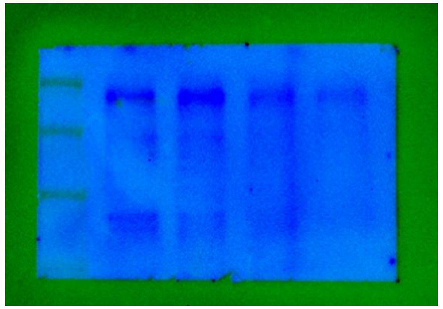

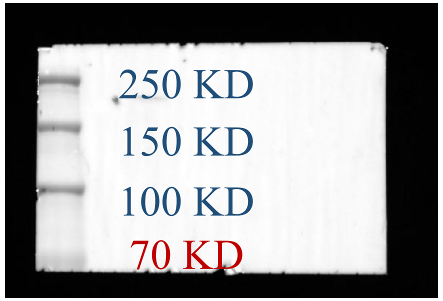

Snail(29 KD)

KYSE-150

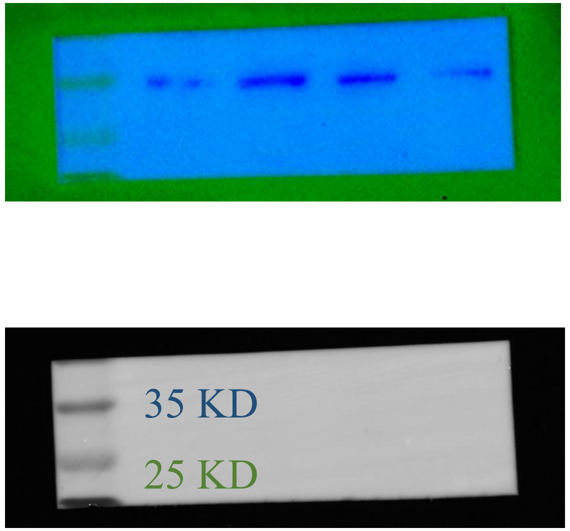

ECA-109

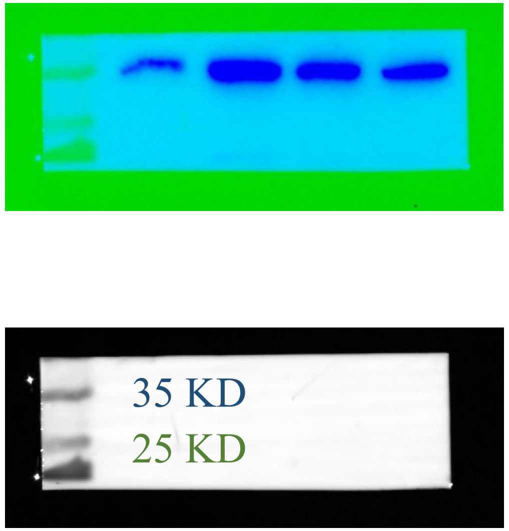

Slug(30 KD)

KYSE-150

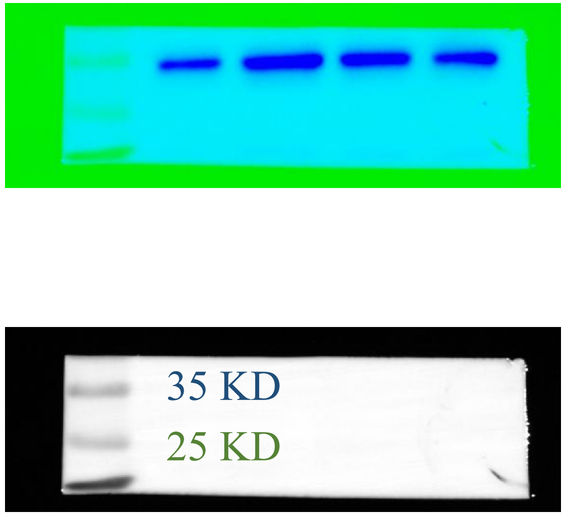

ECA-109

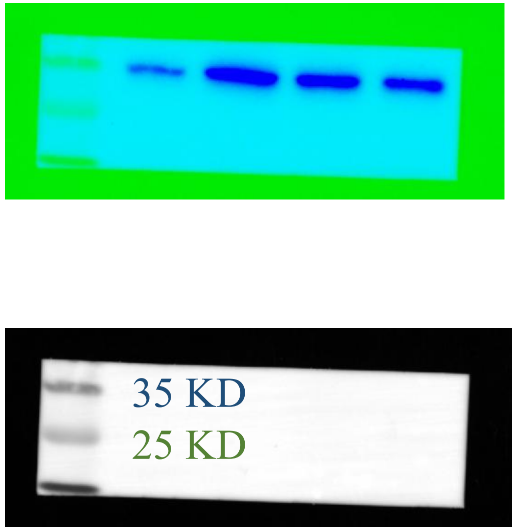

GAPDH(37 KD)

KYSE-150

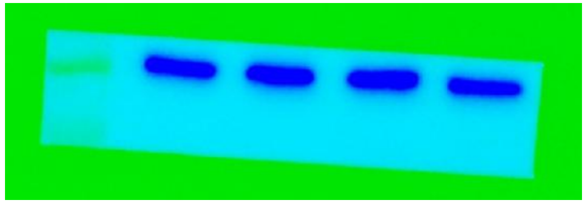

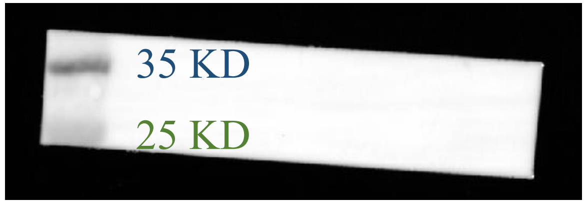

ECA-109

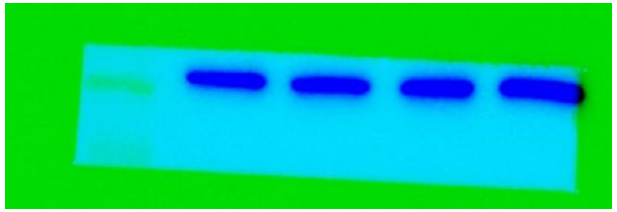

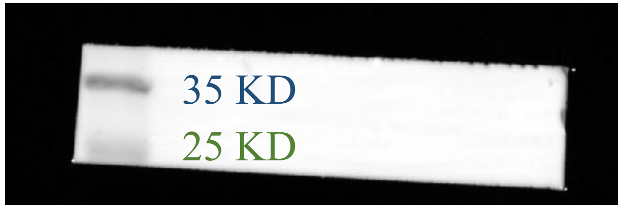

Figure 4. C

HIF-1a(93 KD)

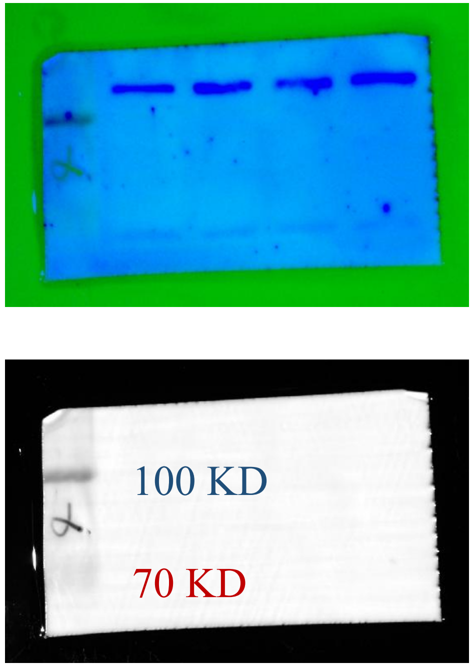

HK II(102 KD)

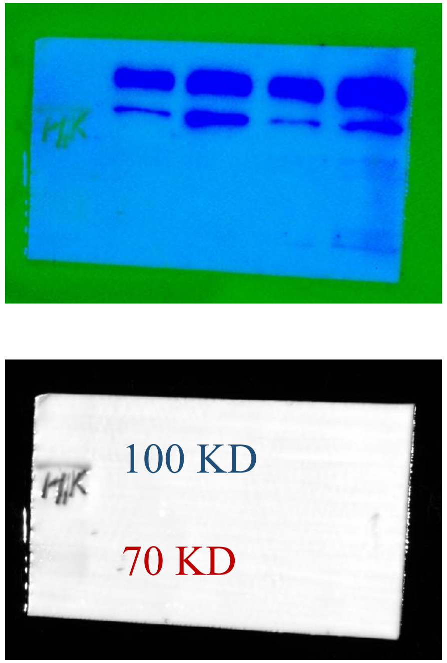

PKM2(60 KD)

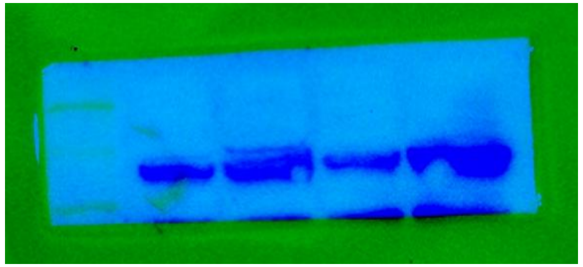

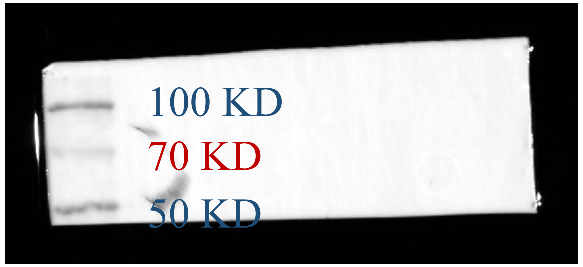

LDHA(37 KD)

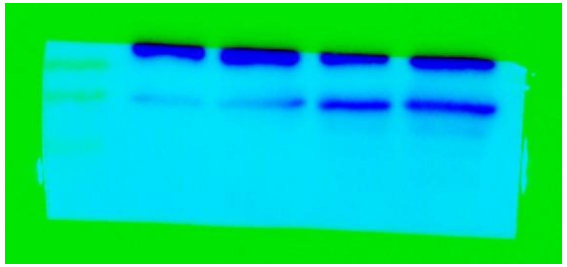

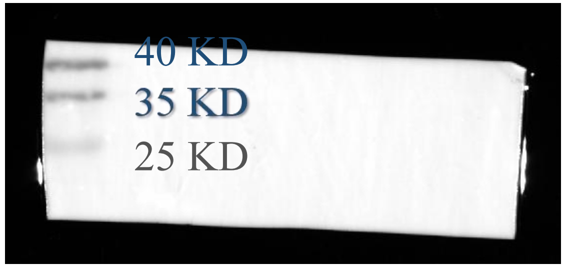

NDUFA13(17 KD)

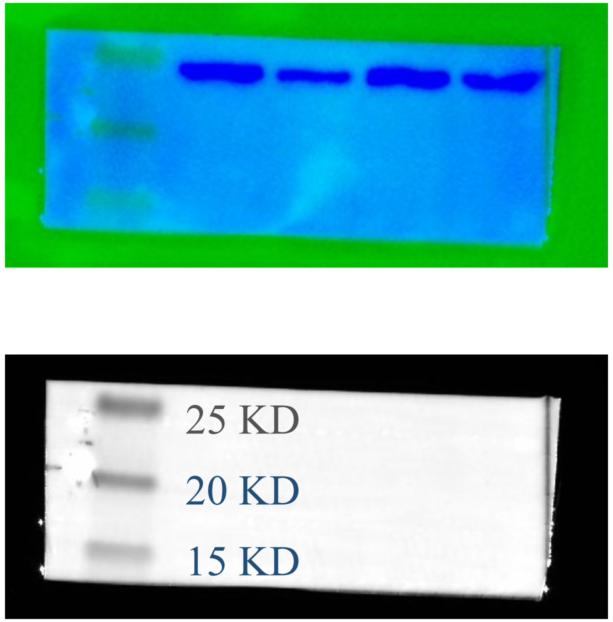

SDHA(70 KD)

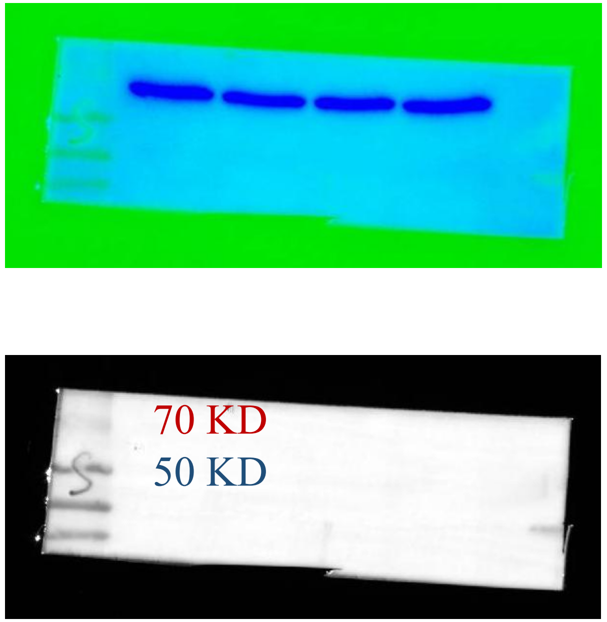

UQCRC2(48 KD)

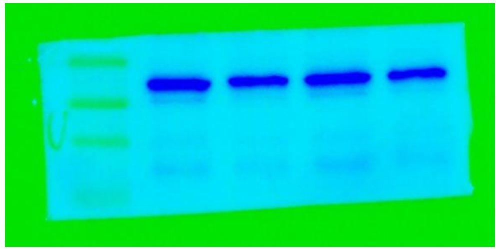

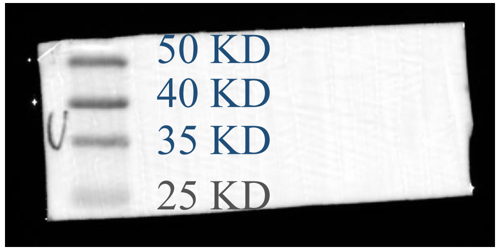

COX IV(17 KD)

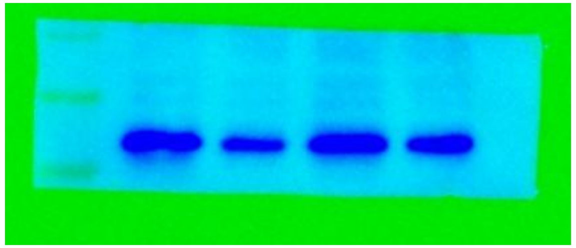

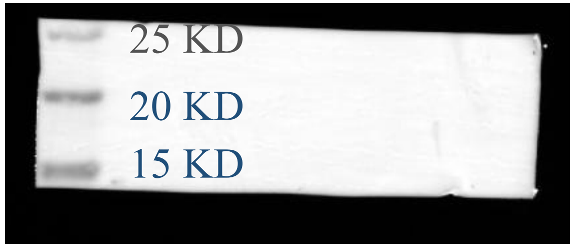

ATP5A1(54 KD)

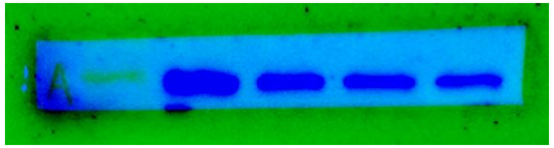

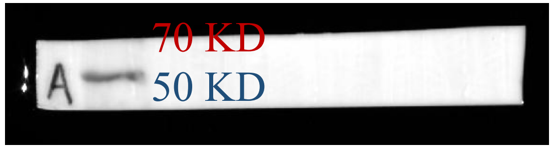

GAPDH(37 KD)

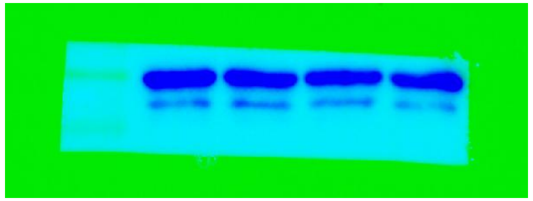

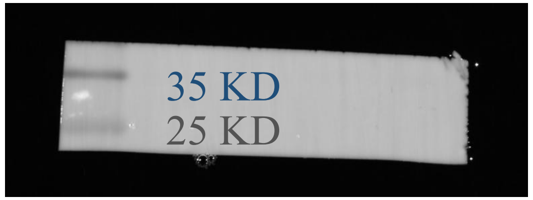

Figure 4. D

HIF-1a(93 KD)

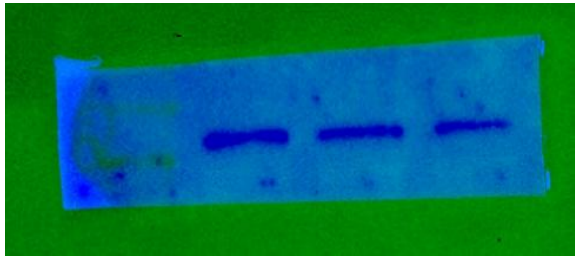

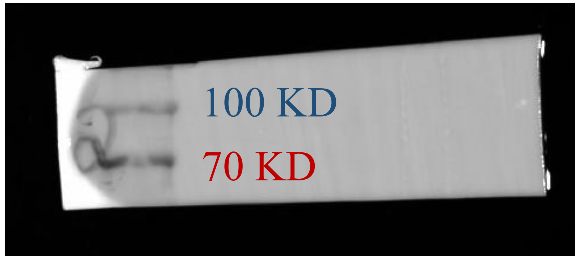

HK II(102 KD)

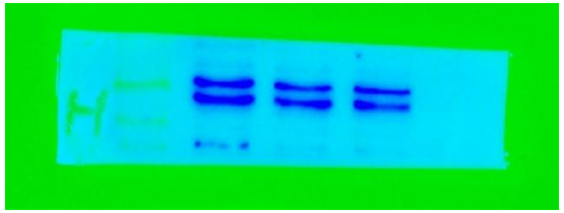

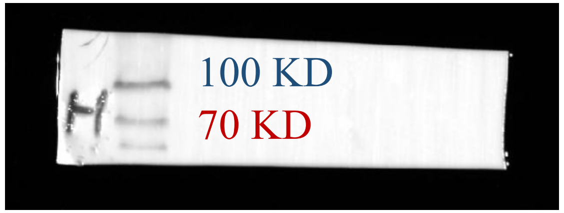

PKM2(60 KD)

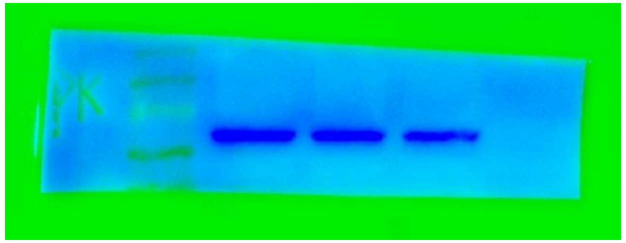

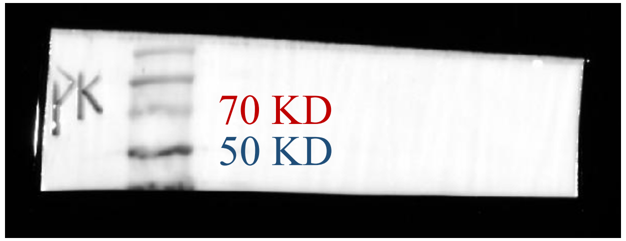

LDHA(37 KD)

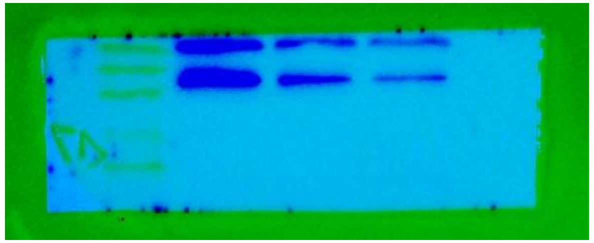

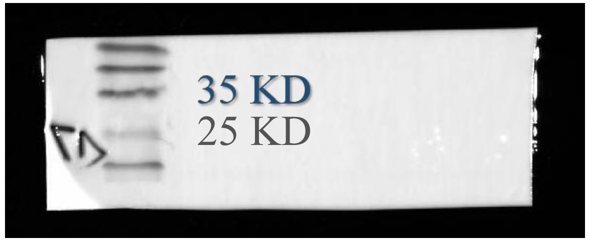

NDUFA13(17 KD)

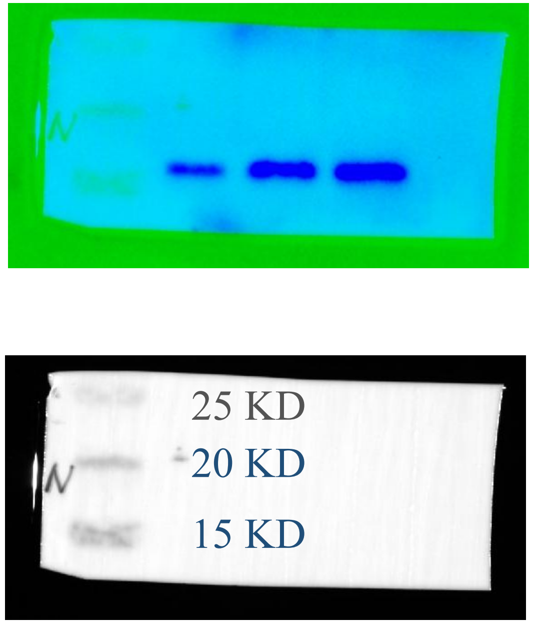

SDHA(70 KD)

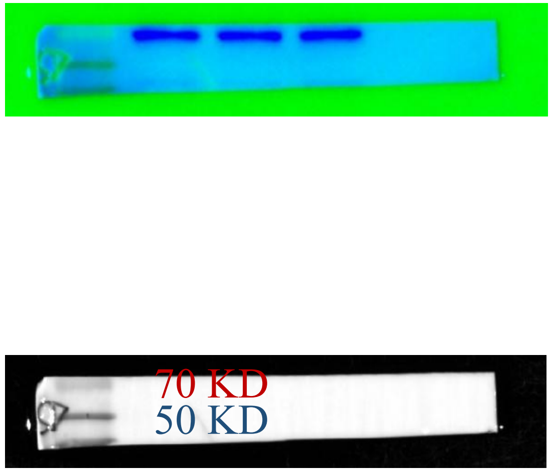

UQCRC2(48 KD)

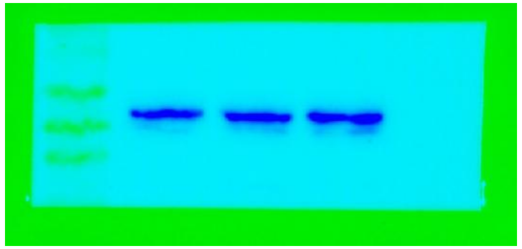

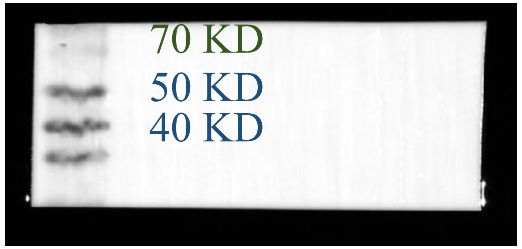

COX IV(17 KD)

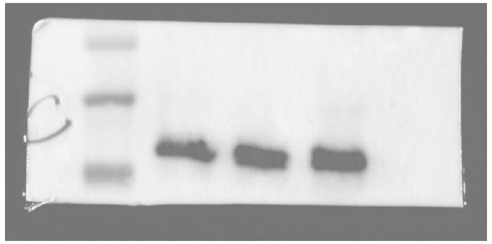

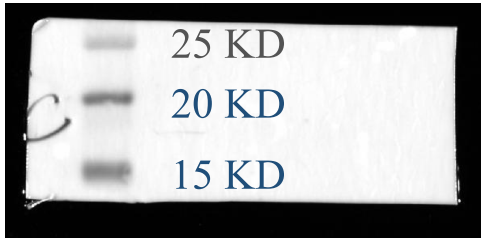

ATP5A1(54 KD)

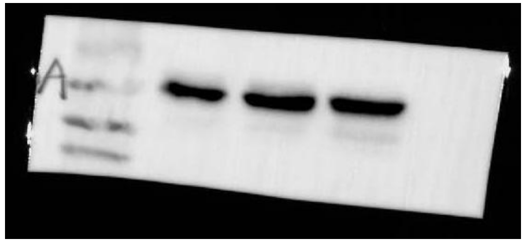

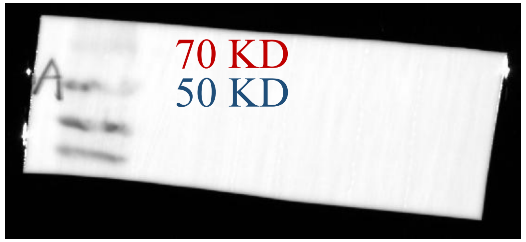

GAPDH(37 KD)

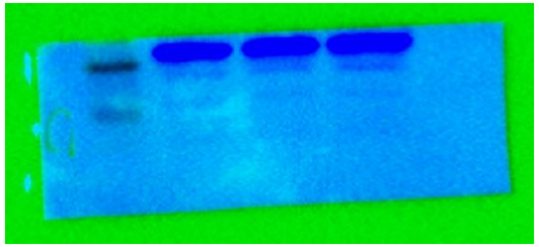

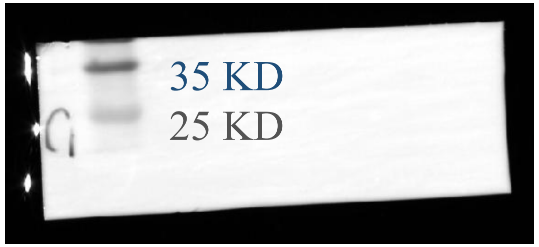

Figure 5. E

HIF-1a(120 KD)

KYSE-150

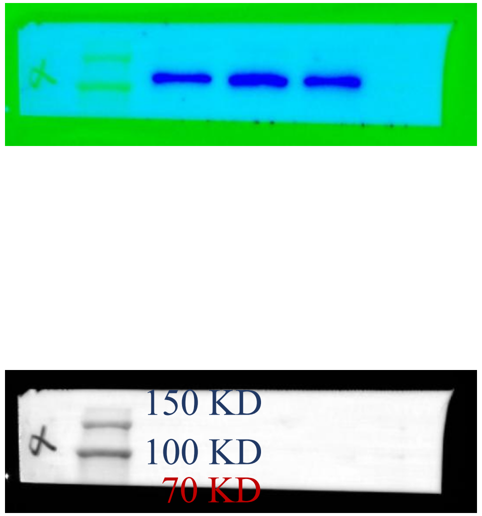

ECA-109

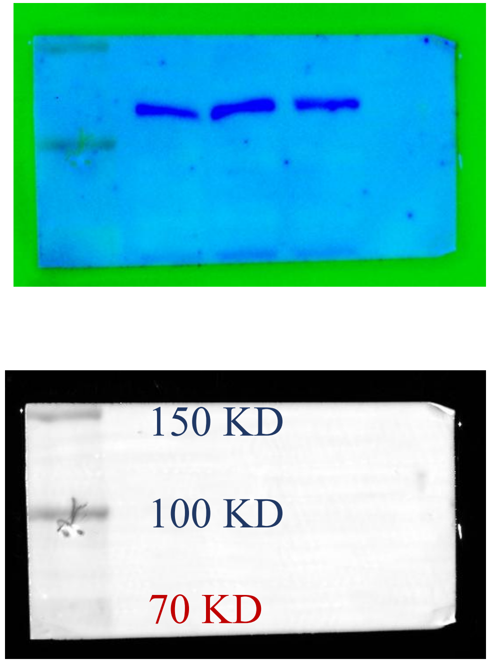

HK II(102 KD)

KYSE-150

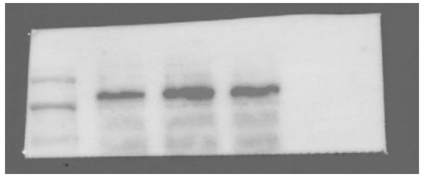

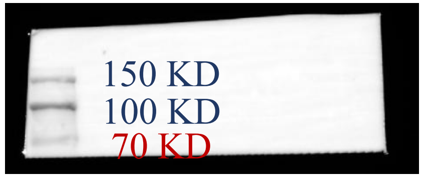

ECA-109

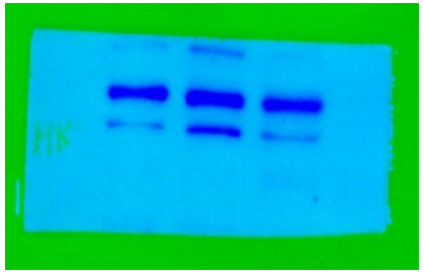

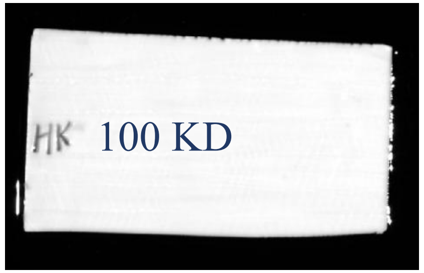

PKM2(60 KD)

KYSE-150

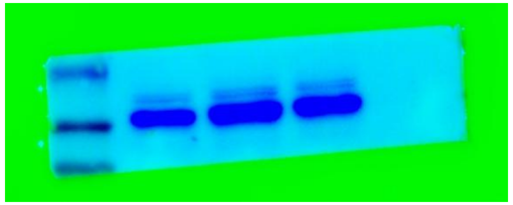

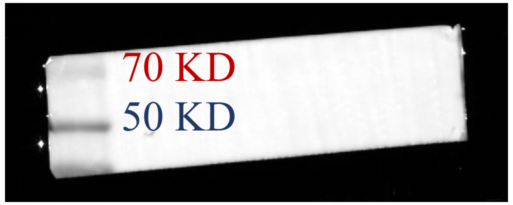

ECA-109

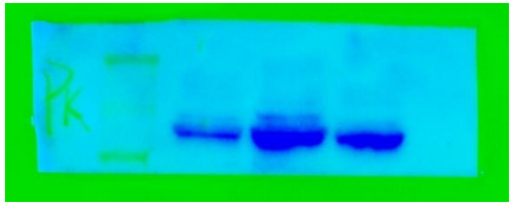

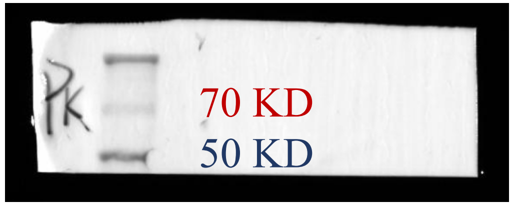

LDHA(37 KD)

KYSE-150

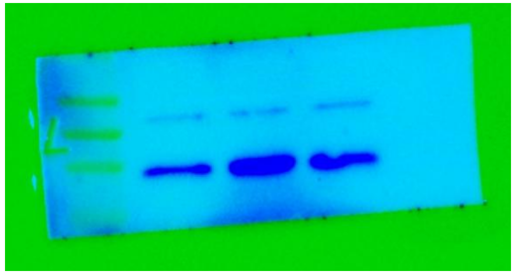

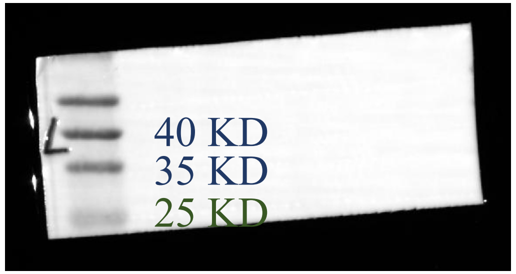

ECA-109

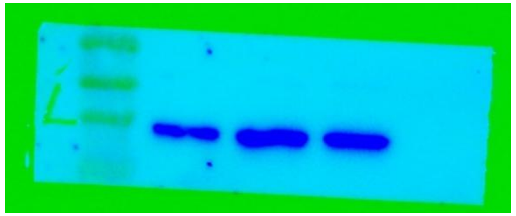

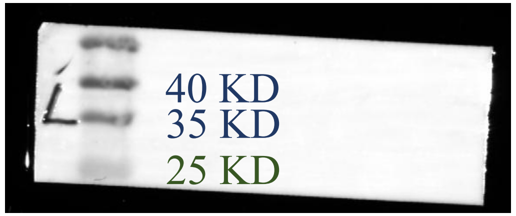

NDUFA13(17 KD)

KYSE-150

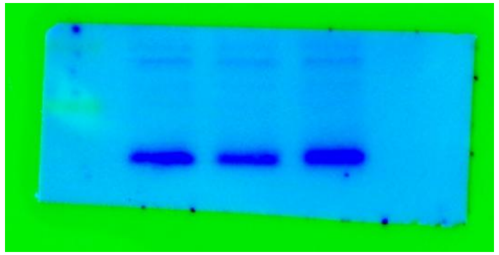

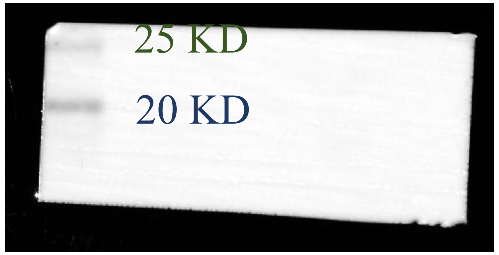

ECA-109

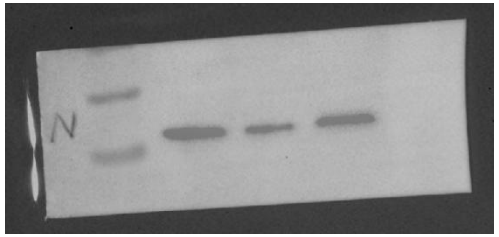

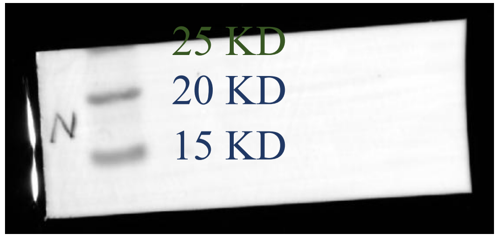

SDHA(70 KD)

KYSE-150

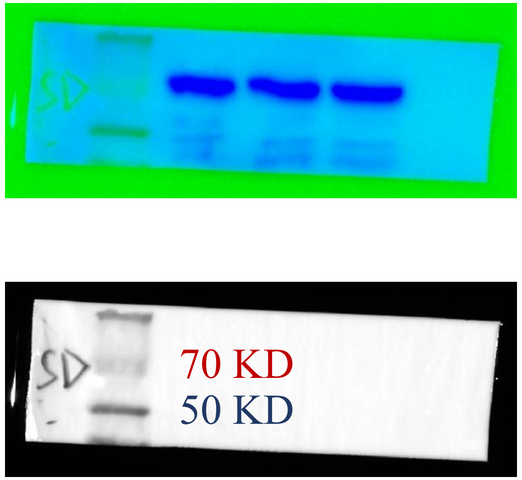

ECA-109

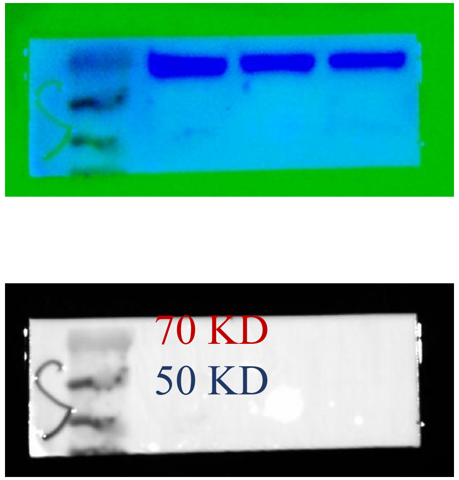

UQCRC2(48 KD)

KYSE-150

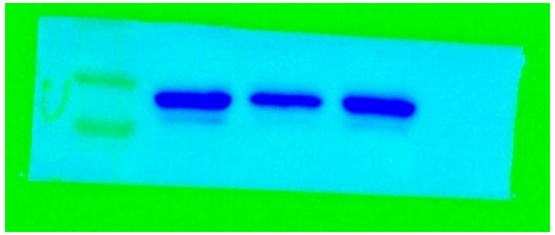

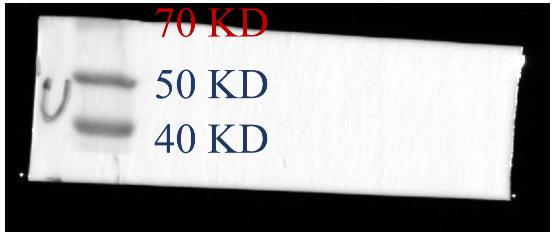

ECA-109

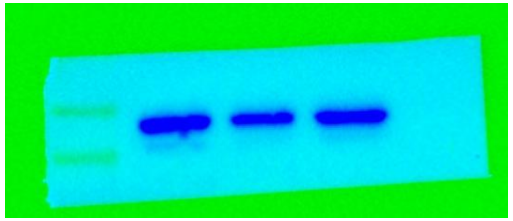

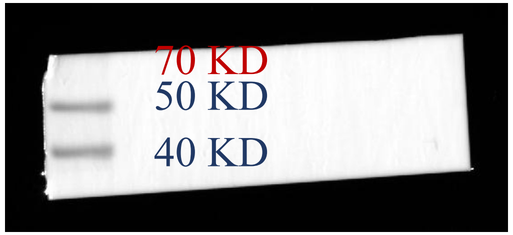

COX IV(17 KD)

KYSE-150

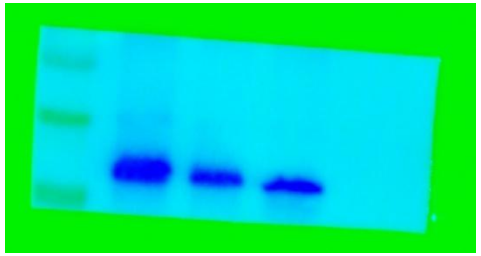

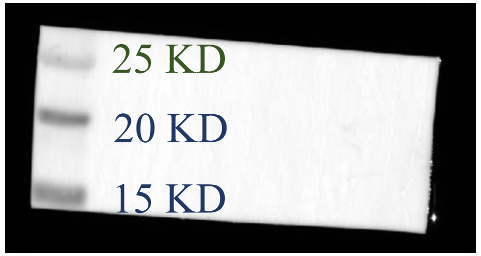

ECA-109

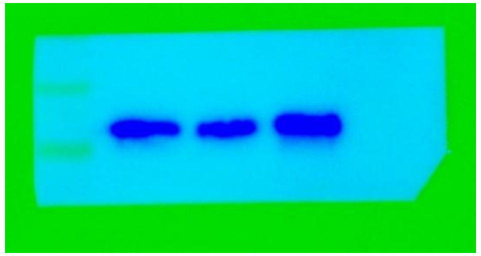

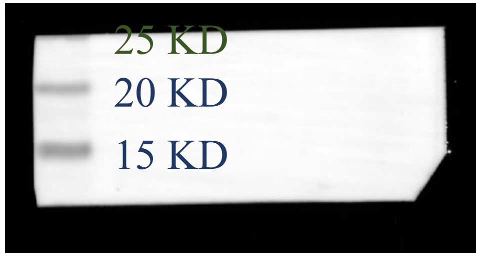

ATP5A1(54 KD)

KYSE-150

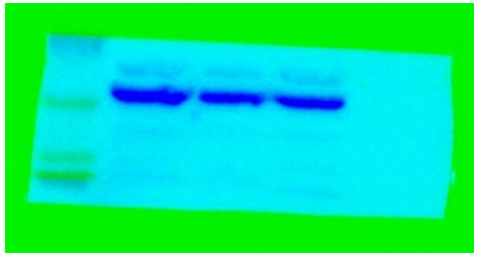

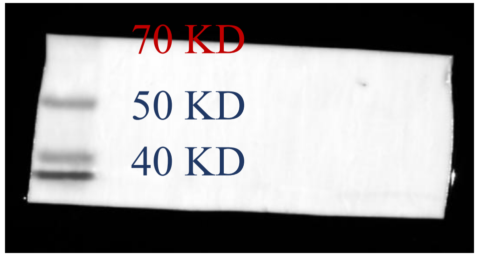

ECA-109

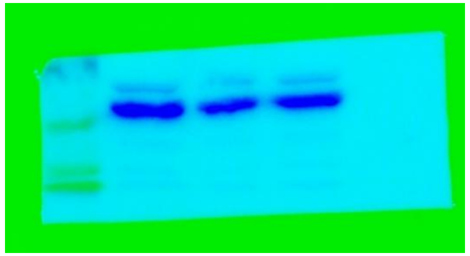

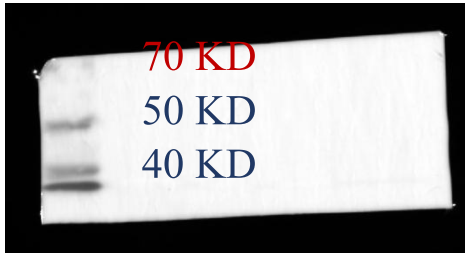

GAPDHA(37 KD)

KYSE-150

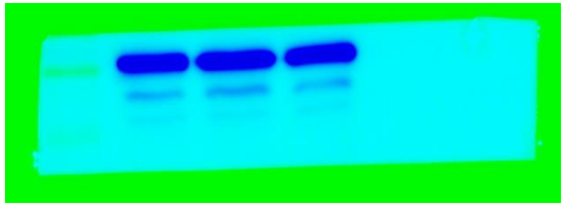

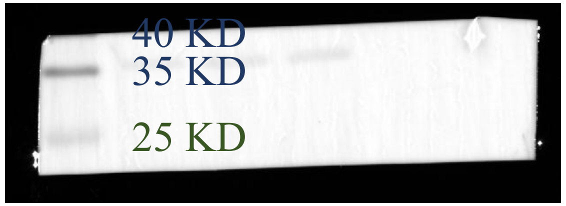

ECA-109

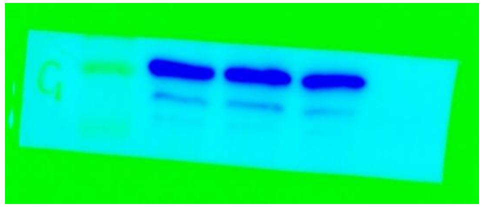

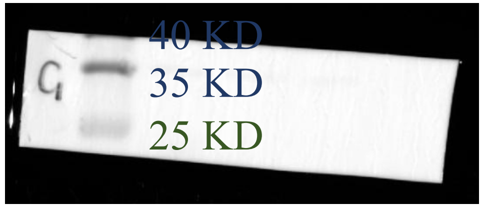

Figure 6. **A**

LDHA(37 KD)

KYSE-150

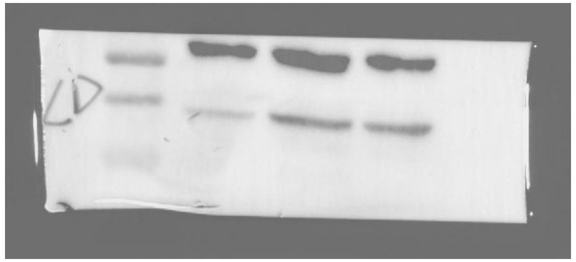

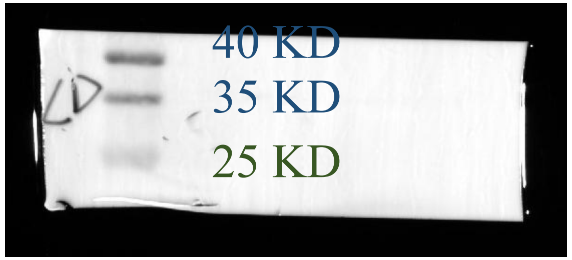

ECA-109

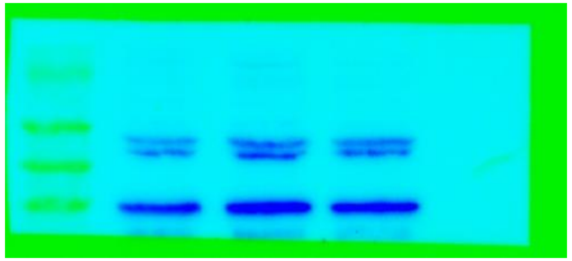

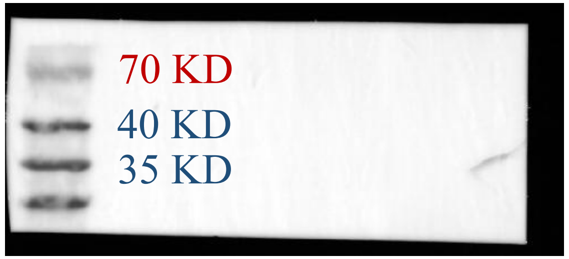

β-actin(45 KD)

KYSE-150

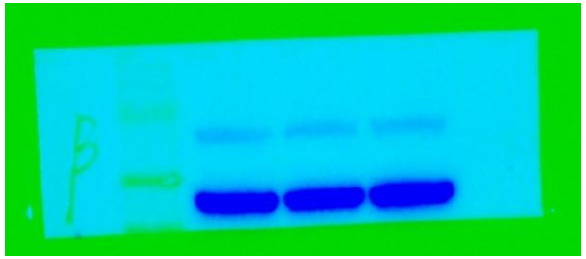

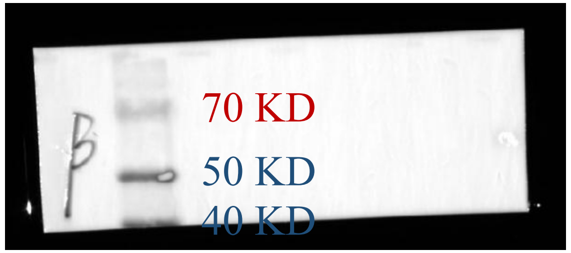

ECA-109

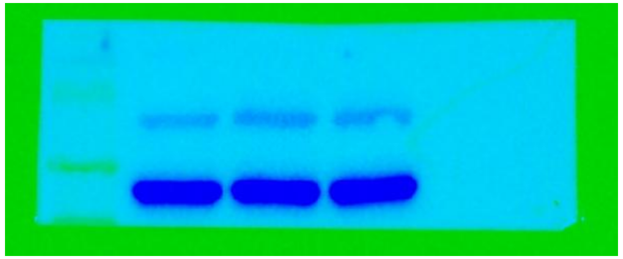

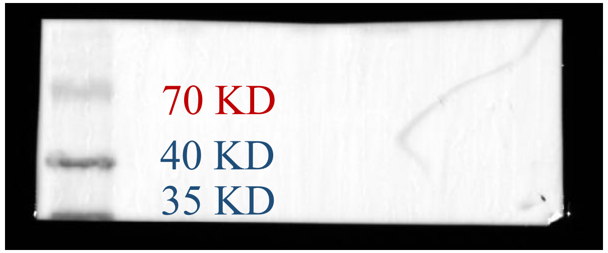

Figure 6. **E**

E-cadherin(135 KD)

KYSE-150

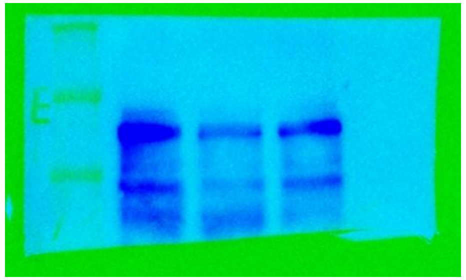

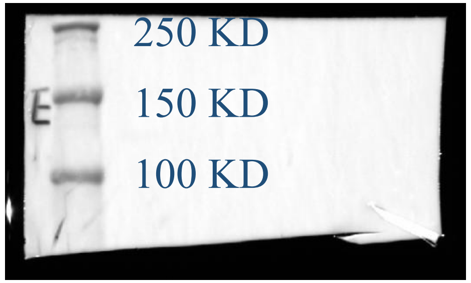

ECA-109

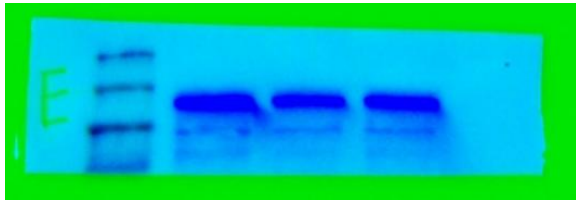

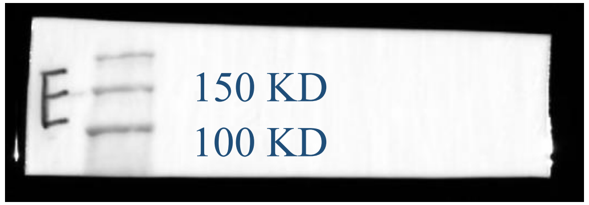

Zeb1(200 KD)

KYSE-150

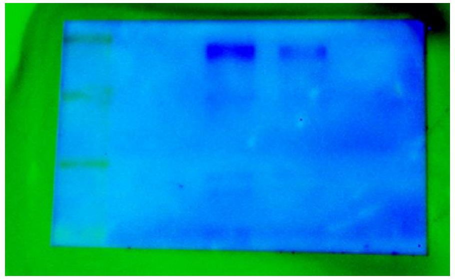

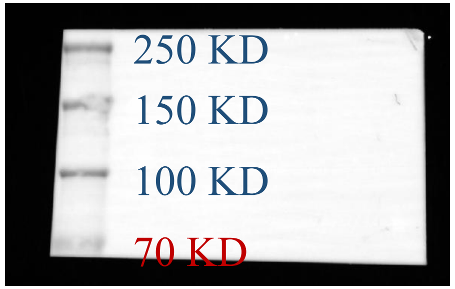

ECA-109

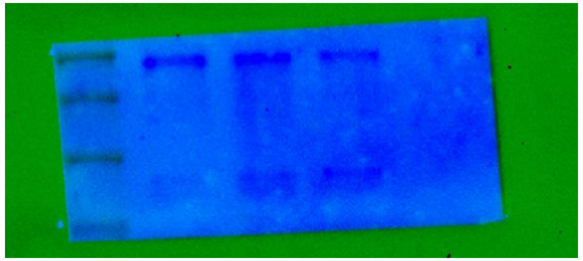

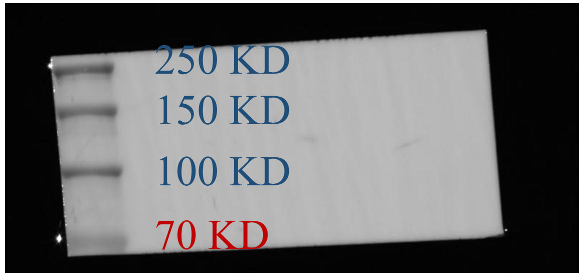

Snail(29 KD)

KYSE-150

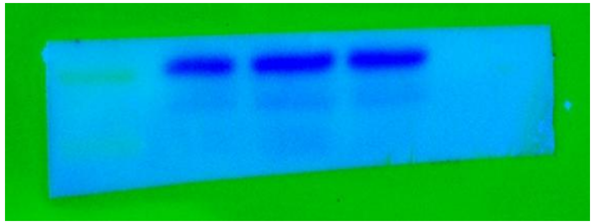

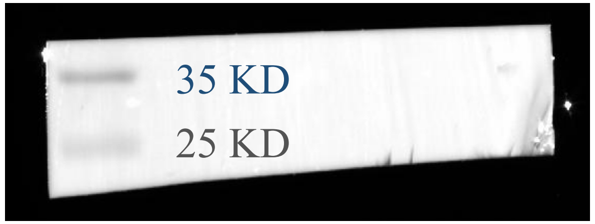

ECA-109

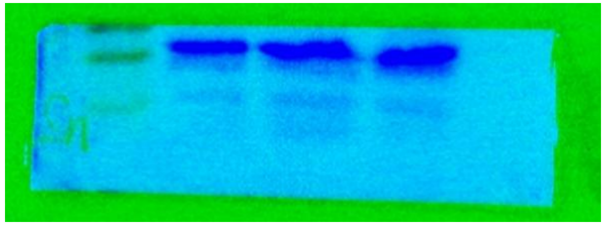

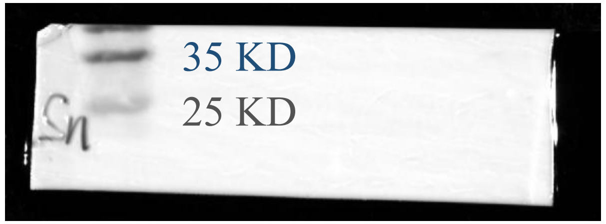

Slug(30 KD)

KYSE-150

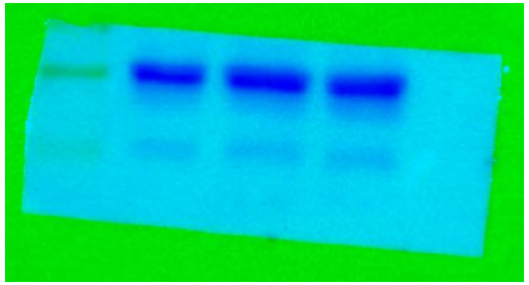

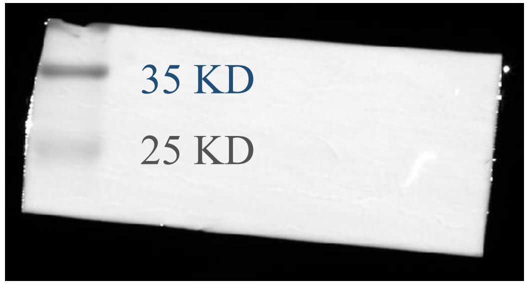

ECA-109

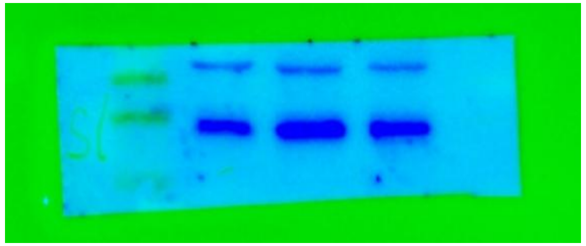

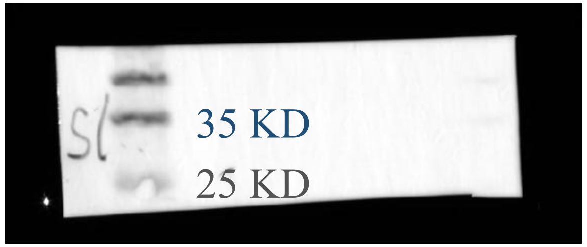

β-actin(45 KD)

KYSE-150

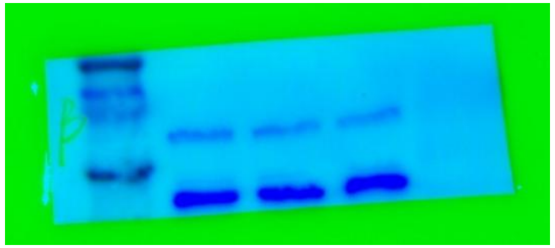

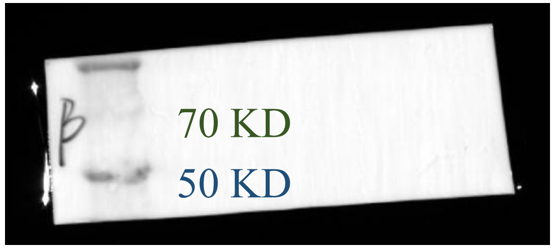

ECA-109

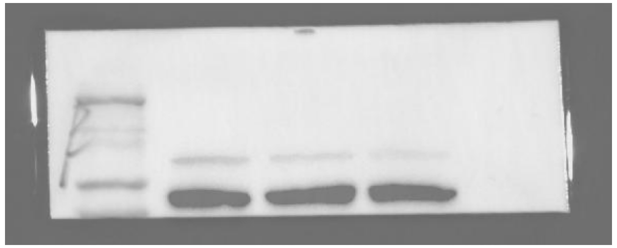

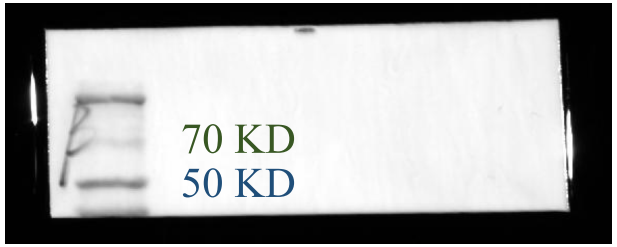

Figure 7. E

HIF-1a(120 kD)

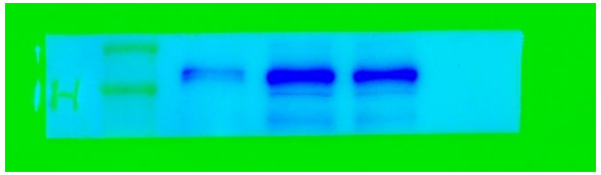

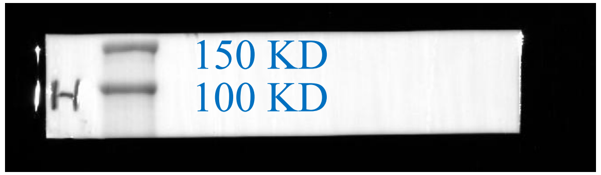

HK II(102 kD)

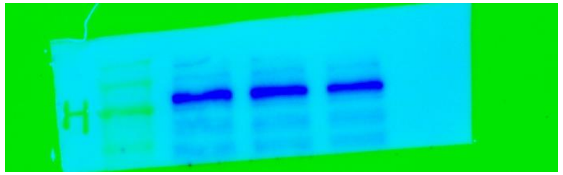

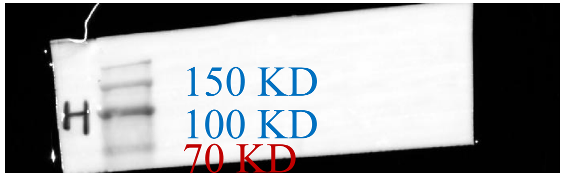

PKM2(60 kD)

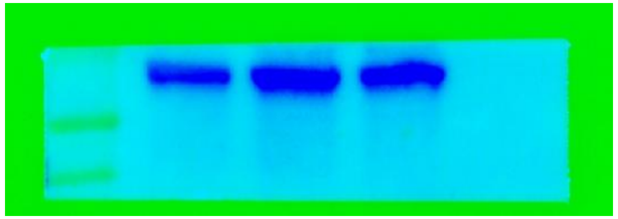

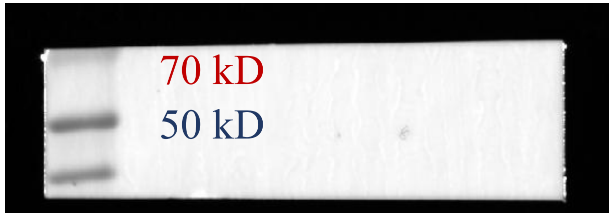

LDHA(37 kD)

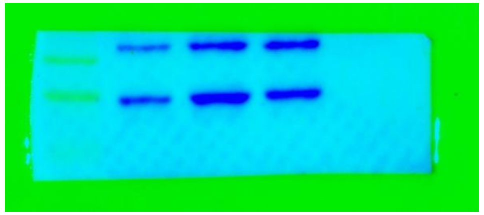

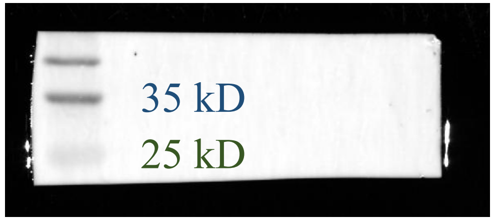

E-cadherin(135 kD)

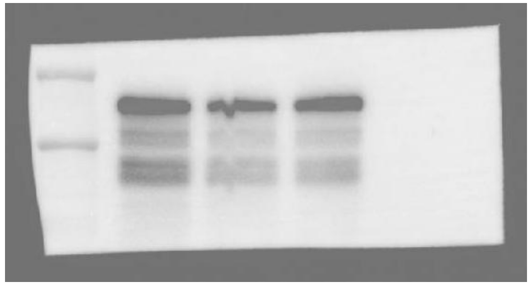

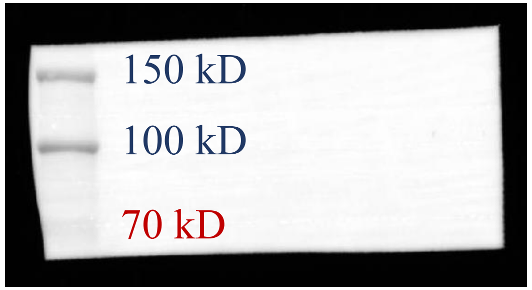

Zeb1(200 kD)

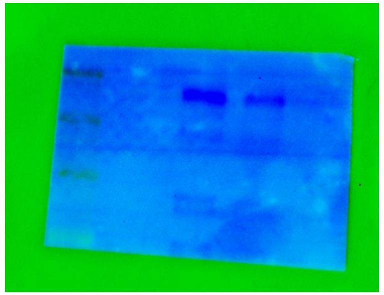

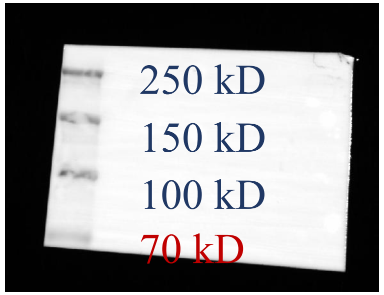

Snail(29 kD)

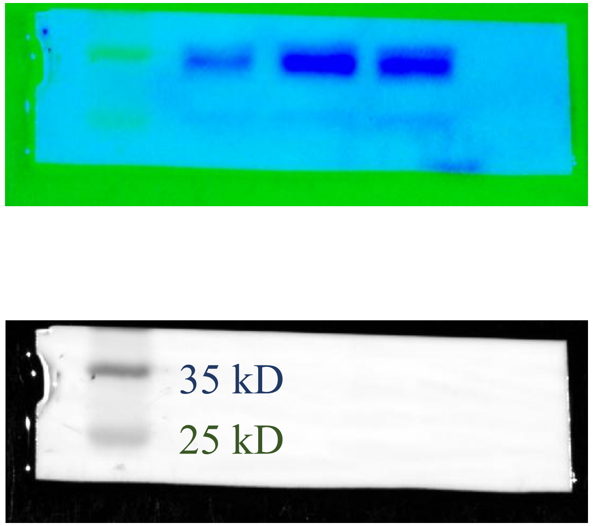

Slug(30 kD)

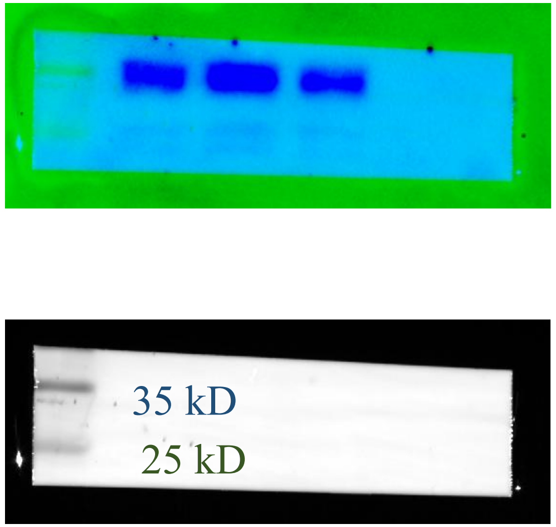

β-actin(42 kD)

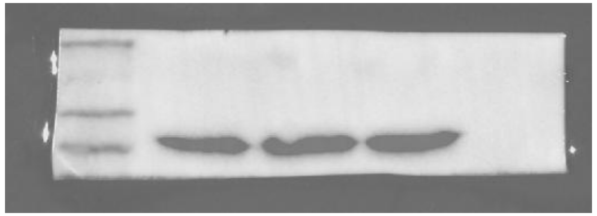

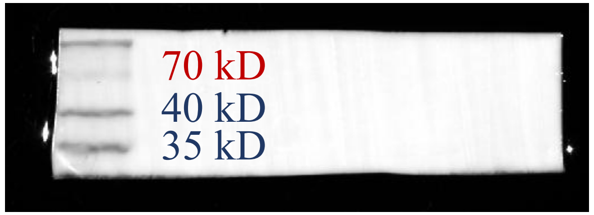

SFigure 1. A

TGF-β

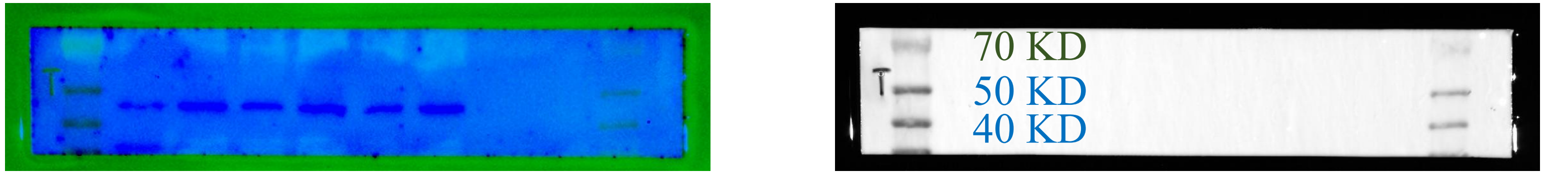

GAPDH

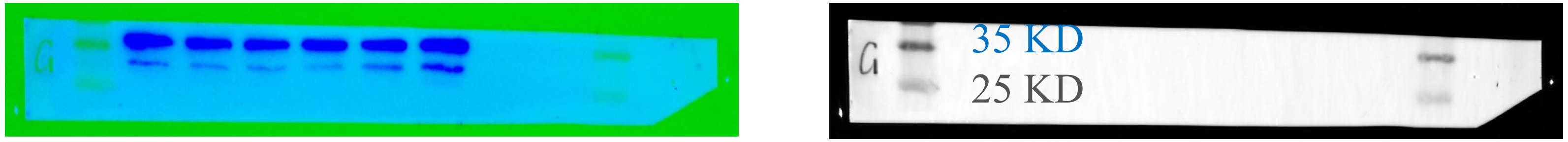

TGF-β

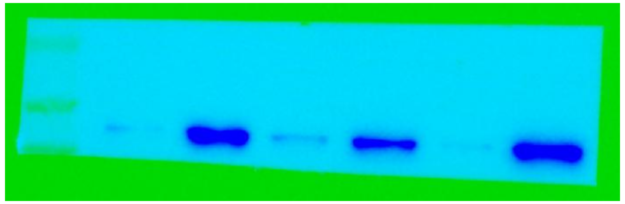

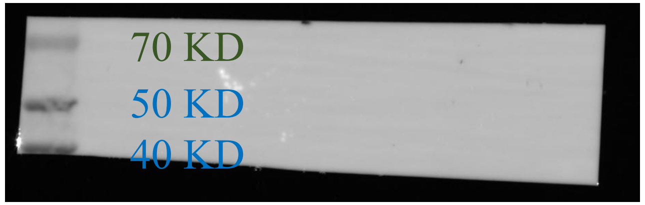

GAPDH

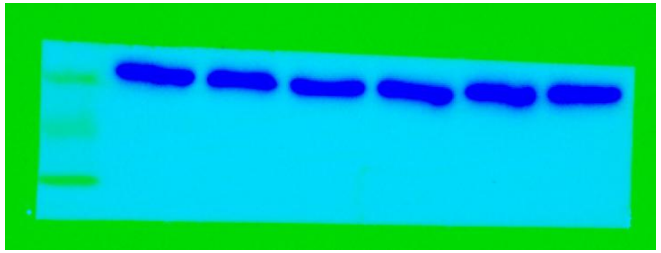

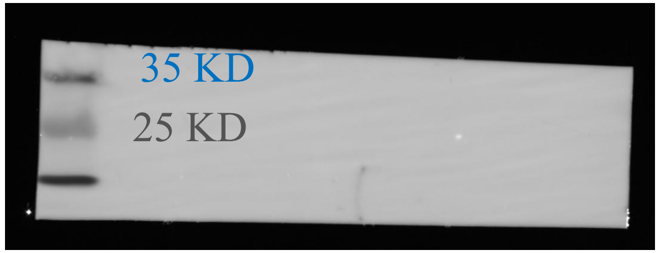

Supplement: Supplementary file 5 — Original Data File [file 41420_2023_1694_MOESM5_ESM.pdf]
